# Supplementary material for: Ketamine restriction correlates with reduced cholestatic liver injury and improved outcomes in critically ill patients with burn injury
Source: JHEP Rep. 2023 Nov 2;6(2):100950. doi: 10.1016/j.jhepr.2023.100950 (PMC10832380; doi:10.1016/j.jhepr.2023.100950)
Supplement: Multimedia component 1 [file mmc1.pdf]

Ketamine restriction correlates with reduced cholestatic liver injury and improved outcomes in critically ill burn patients

Christian De Tymowski, François Dépret, Emmanuel Dudoignon, Nabila Moreno, Anne-Marie Zagdanski, Kyann Hodjat, Benjamin Deniau, Alexandre Mebazaa, Matthieu Legrand, Vincent Mallet for the Keta-Cov research group

Table of contents

List of investigators 2

Fig. S1 3

Fig. S2 4

Fig. S3 5

Table S1 6

Table S2 9

Table S3 11

List of investigators

The Keta-Burn research group

Christian De Tymowski[1,2,3], François Dépret[1,3,4,5,6], Emmanuel Dudoignon[3], Nabila Moreno[7], Anne-Marie Zagdanski[8], Kyann Hodjat[3], Benjamin Deniau[1,3,4,6], Maxime Coutrot,[3] Mourad Benyamina,[3] Thibault Michel,[3] Alexandru Cupaciu,[3] Alexandre Pharaboz,[3] Maurice Mimoun,[3] Marc Chaouat,[3] Kevin Serror,[3] Benoit Plaud,[3] Maïté Chaussard,[3] Lucie Guillemet,[3] Alexandre Mebazaa[1,3,5,7], Matthieu Legrand[6,9], Vincent Mallet[1,3,10]

1. Université Paris Cité, F-75006, Paris, France
2. Department of Anaesthesiology and Surgical Intensive Care Unit, Groupe Hospitalier Bichat Claude Bernard, DMU PARABOL, Assistance Publique-Hôpitaux de Paris, Paris, France.
3. AP-HP.Nord, Groupe Hospitalier Saint Louis Lariboisière, DMU PARABOL, Département d'anesthésie réanimation et centre de traitement des brûlés, Paris, France
4. Institut National de la Santé et de la Recherche Médicale (INSERM), INSERM UMR-S 942 Mascot, Lariboisière Hospital, Paris, France
5. INI-CRCT Network, Nancy, France
6. FHU PROMICE, Paris, France
7. AP-HP.Nord, Groupe Hospitalier Saint Louis Lariboisière, Laboratoire de Biochimie, Paris, France;
8. AP-HP.Nord, Groupe Hospitalier Saint Louis Lariboisière, Département de radiologie, Paris, France;
9. Department of Anesthesia and Peri-operative Care, Division of Critical Care Medicine, University of California, San Francisco, USA
10. Assistance Publique — Hôpitaux de Paris (AP—HP), Groupe Hospitalier Cochin Port Royal, DMU Cancérologie et spécialités médico-chirurgicales, Service d'Hépatologie, Paris, France

Supplementary figures

Fig. S1: Covariate balance in the propensity matched samples

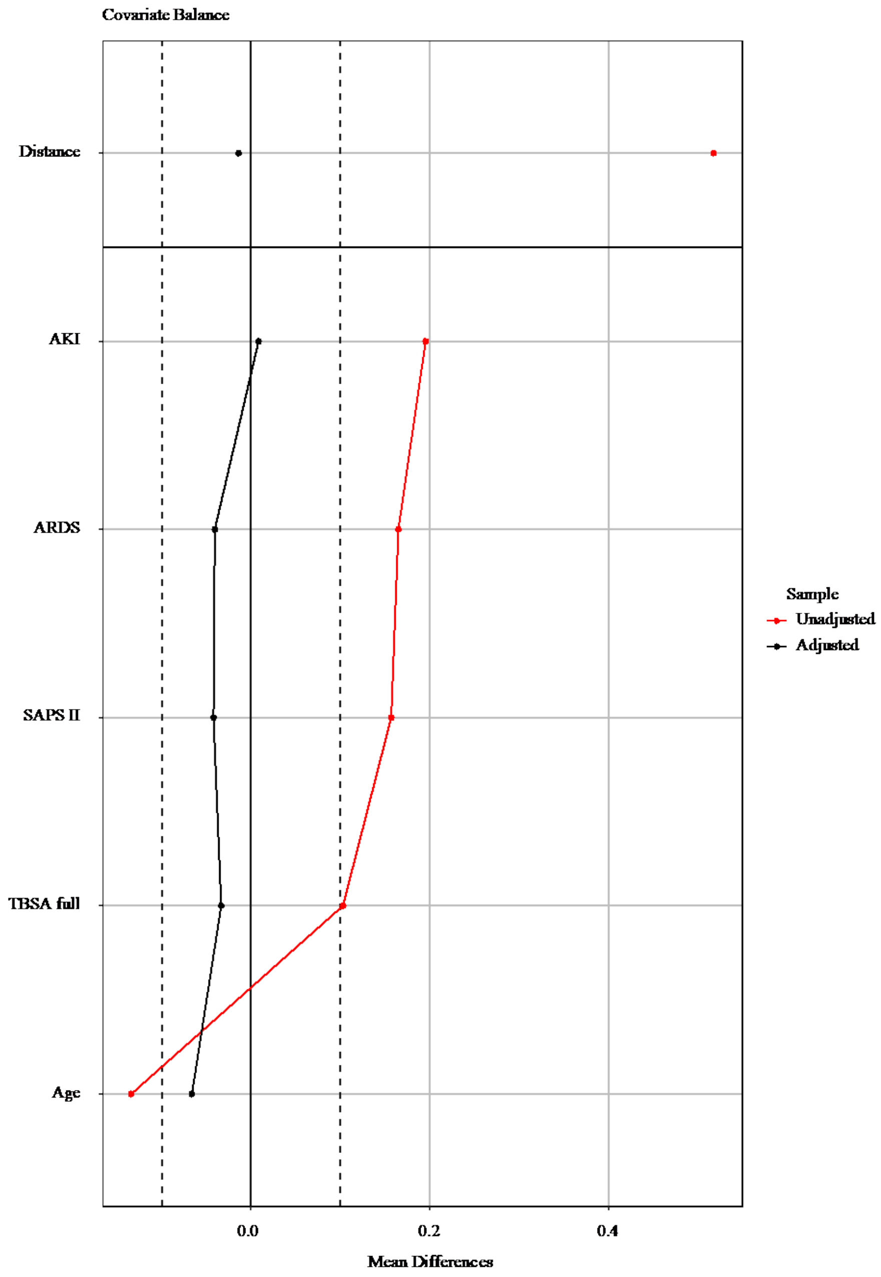

Matching was performed using the MatchIt package (Ho, Imai, King, & Stuart, *2011*) , and covariate balance was assessed using Cobalt Greifer, N. (2023). cobalt: Covariate Balance Tables and Plots. R package version 4.4.1.9003. AKI: acute kidney injury; ARDS: Acute Respiratory Distress Syndrome FTB BSA: full thickness burned surface area; SAPS II: simplified acute physiology score II; SOFA: Sequential Organ Failure Assessment score

Fig. S2: Liver test evolution over the study period

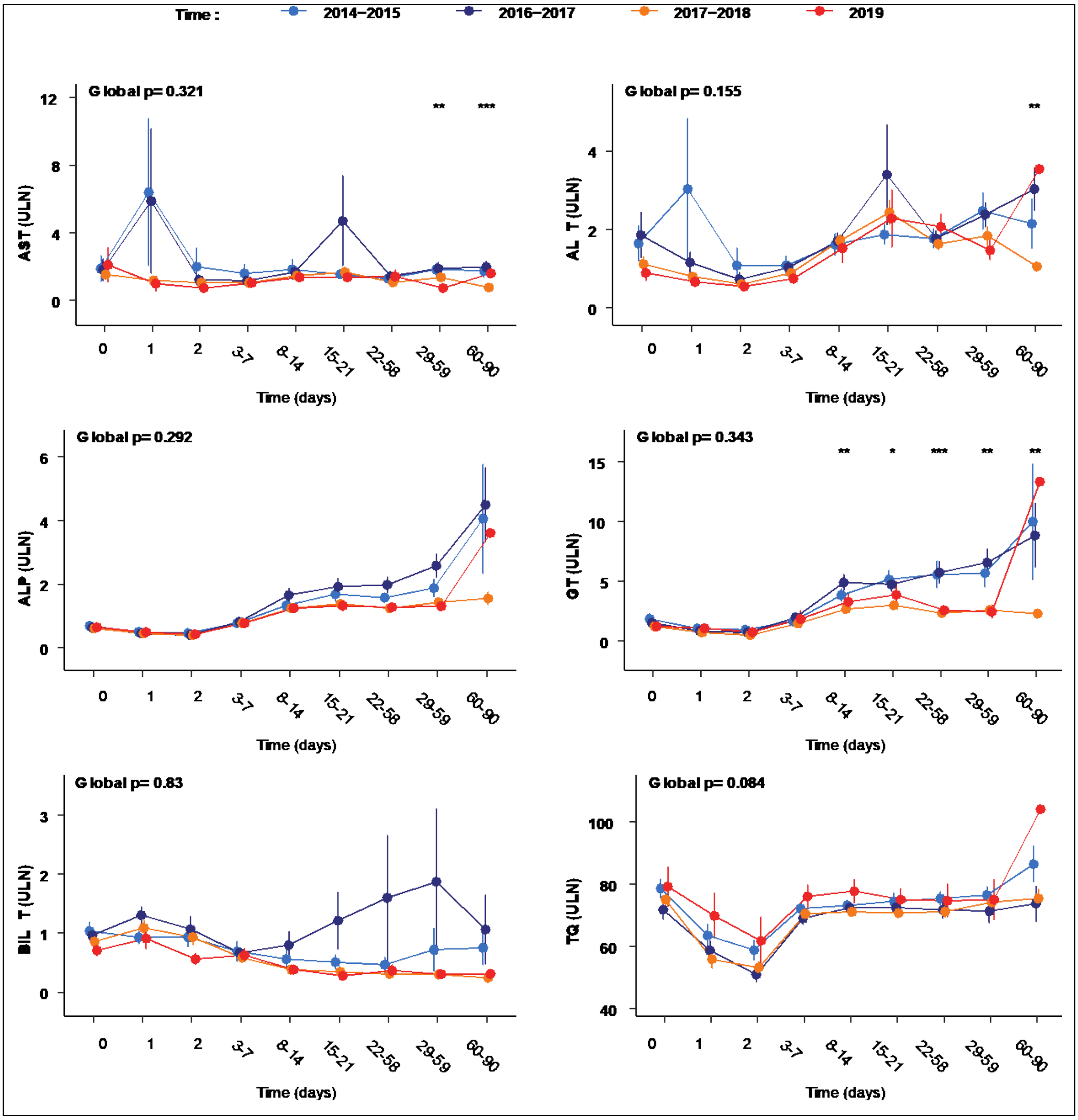

Global p-values were computed with mixed models. The comparison of the four groups at each time point was performed with Kruskal-Wallis tests, with statistical significance indicated as follows: (ns, p > 0.05; * p < 0.05; ** p < 0.01; *** p < 0.001; **** p < 0.0001). The time periods 2014-2015 and 2016-2017 corresponded to the ketamine-liberal period from December 2014 to the end of March 2017 when ketamine prescription was 'liberally' used for maintenance sedation (≥ 1mg/kg/h). The time periods 2017-2018 and 2019 corresponded to the ketamine-restricted period, from April 2017 to June 2019, when ketamine was only used as a second-line co-analgesic drug with a capped dose (< 0.015mg/kg/h). Note: ALP stands for alkaline phosphatase; ALT for alanine aminotransferase; AST for aspartate aminotransferase; BILT for total bilirubin; and GGT for gamma-glutamyl transferase.

Fig. S3: Cholangitis in a patient exposed to ≥ 10,000 mg ketamine

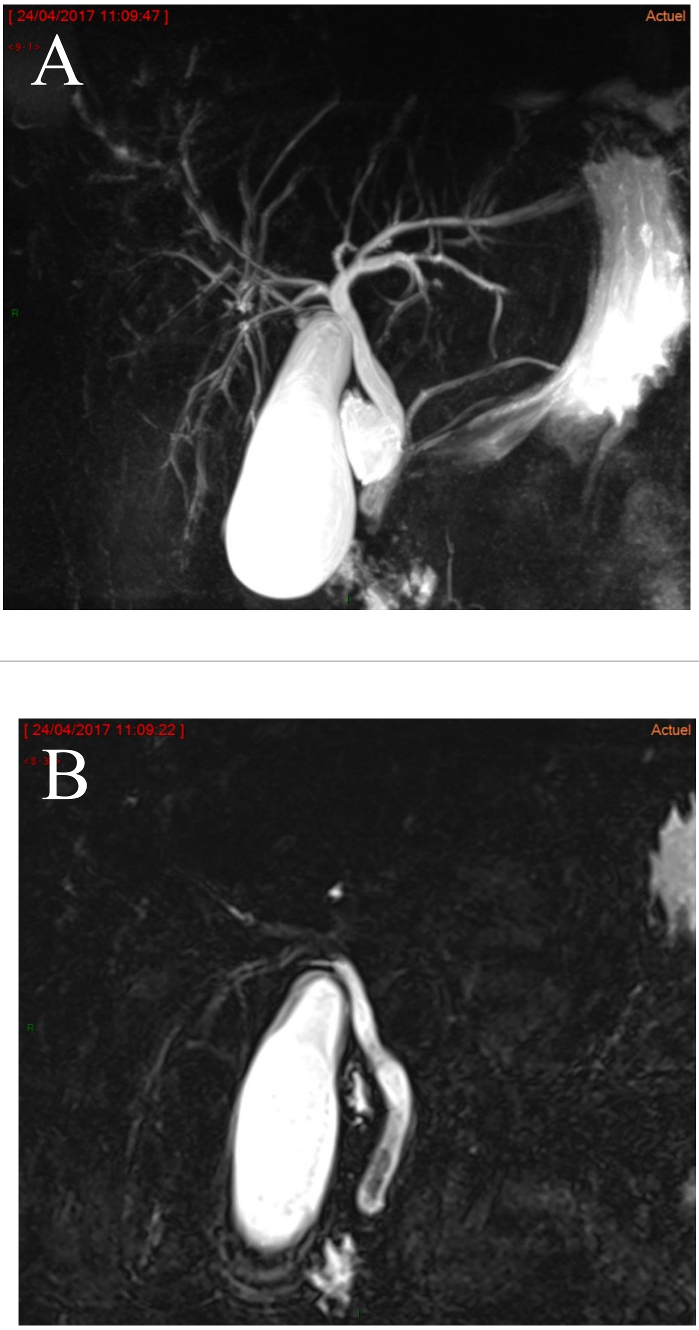

A MR Cholangiopancreatography with a maximum intensity projection (MIP) reconstruction from a 3D thin section showing dilation of the bile ducts;

B Thin sections of the MRI showing biliary casts in the common bile duct

Table S1: Characteristics of patients with cholestatic liver injury

Time Period	Sex	Age (years)	TBSA (%)	ket doses (mg)	Ket days	Time to CLI (days)	Time ket-CLI (days)	Infection	Delay Infection-CLI (days)	Antibiotics	Antifungal	Antiviral	CT-scan	RECAM score	RECAM probability	ALP ≥ grade 3	Long term liver injury
**Liberal**	M	36.2	30	0	0	17	-	y	5	Piperacillin Tazobactam			Normal	-	-	n	
**Liberal**	M	26.3	57	1,441	5	14	-30	y	6	Piperacillin Tazobactam				-11	Unlikely	y	
**Liberal**	M	55.3	16	0	0	9	-	n	-	Cefepime				-	-	y	
**Liberal**	F	84	59	0	0	1	-	n	-					-	-	n	
**Liberal**	F	64.1	57	17,826	46	10	10	n	-	Piperacillin Tazobactam			Normal	-1	Possible	y	UPC
**Liberal**	M	43.3	31	42,206	16	19	18	y	9		Amphotericin B		Normal	3	Possible	n	UPC
**Liberal**	M	69.9	16	5,996	4	19	11	y	12	Amoxicillin Clavulanate			Normal	9	Highly probable	n	
**Liberal**	F	50.7	40	43,775	34	47	46	y	3	Piperacillin Tazobactam	Amphotericin B		BDD	5	Probable	y	UPC/Biliary sepsis
**Liberal**	F	69.1	70	29,469	13	12	12	y	5		Amphotericin B		Normal	9	Highly probable	y	
**Liberal**	M	49.6	60	21,232	6	10	10	y	1		Amphotericin B		Normal	3	Possible	n	
**Liberal**	M	47.5	62	10,722	23	5	5	y	1	Cefepime			Normal	4	Probable	y	UPC
**Liberal**	M	53.1	45	16,879	11	7	6	n	-				Normal	6	Probable	n	
**Liberal**	M	40.1	55	9,451	16	32	31	y	1	Cefepime			Normal	5	Probable	n	
**Liberal**	M	25.3	70	28,289	32	14	12	y	3		Caspofungine		BDD; Multiple liver abscess	5	Probable	y	
**Liberal**	M	57	58	12,173	38	68	67	y	7	Ceftazidime			Normal	3	Possible	n	

**Liberal**	M	56	80	15,490	13	8	7	y	4	Piperacillin Tazobactam			Normal	-4	Unlikely	y	UPC
**Liberal**	M	51	70	9,403	6	9	9	y	0	Amoxicillin Clavulanate			Liver dysmorphia	2	Possible	y	
**Liberal**	F	25.8	65	28,279	10	23	23	y	7	Meronem			Normal	3	Possible	n	UPC
**Liberal**	M	32.7	86	10,421	52	11	11	y	4	Meronem			Normal	5	Probable	y	UPC/ Biliary sepsis
**Liberal**	M	68.3	20	20,121	7	8	3	y	0	Amoxicillin Clavulanate			Normal	2	Possible	y	
**Liberal**	M	35.9	83,5	39,135	22	16	16	y	4		Amphotericin B	Acyclovir	Normal	5	Probable	n	UPC
**Liberal**	F	68.1	37	41,339	17	11	10	y	6	Piperacillin Tazobactam		Acyclovir		7	Probable	y	
**Liberal**	F	72.3	20	20	1	10	7	y	5				Normal	4	Probable	n	
**Liberal**	F	77.5	30	17,219	29	19	18	y	4	Piperacillin Tazobactam			BDD	5	Probable	y	UPC/ Biliary sepsis
**Liberal**	F	55.8	35	4,525	35	5	4	n	-					0	Possible	y	UPC
**Liberal**	F	65.2	9	0	0	1	-	n	-					-	-	n	
**Liberal**	F	26.9	50	1,132	11	16	16	y	6	Piperacillin Tazobactam			Normal	7	Probable	y	
**Restricted**	M	60.6	7	130	3	14	6	y	6	Amoxicillin Clavulanate			BDD	1	Possible	n	
**Restricted**	M	55.7	39	0	0	37	-	y	8	Piperacillin Tazobactam	Fluconazole		Normal	-	-	n	
**Restricted**	M	26.8	72	130	4	4	-3	n	2	Piperacillin Tazobactam			Normal	-11	Unlikely	y	
**Restricted**	M	47.7	30	23	2	13	-4	y	3	Cefepime				-11	Unlikely	n	
**Restricted**	M	29.3	20	250	4	8	6	y	3	trimethoprim sulfamethoxazole			Normal	8	Highly probable	n	
**Restricted**	F	49.4	7	50	1	4	0	y	5	Amoxicillin Clavulanate			Normal	-1	Possible	n	

**Restricted**	M	16.6	38	0	0	29	-	y	10	Cefotaxime				-	-	n	

Note: BDD: bile duct dilatation; CLI: cholestatic liver injury; Ket: Ketamine; n=no, UPC: unexplained prolonged cholestatic liver injury; y=yes

Table S2: Characteristics of patients by day-90 mortality				
**Characteristic**	Overall, N = 278 (100%)[1]	Day-90 mortality		p-value[2]
		No death, N = 221 (79%)[1]	Death, N = 57 (21%)[1]	
**Ketamine dose reduction period**				0.031
Before reduction	155 (55.8%)	116 (52.5%)	39 (68.4%)	
After reduction	123 (44.2%)	105 (47.5%)	18 (31.6%)	
**Cholestatic liver injury**	34 (12.2%)	20 (9.0%)	14 (24.6%)	0.001
**Grade ≥ 3 ALP elevation**	25 (9.0%)	17 (7.7%)	8 (14.0%)	0.14
**DILI cholestasis**	106 (38.1%)	85 (38.5%)	21 (36.8%)	0.8
**Cholestasis**	174 (62.6%)	133 (60.2%)	41 (71.9%)	0.10
**Male sex**	176 (63.3%)	144 (65.2%)	32 (56.1%)	0.2
**Age, years**	50.7 (31.5, 67.3)	45.7 (29.7, 61.4)	65.2 (51.0, 81.5)	<0.001
**Body mass index, Kg/m2**	25.1 (22.9, 28.7)	24.8 (22.9, 28.1)	26.1 (22.5, 29.8)	0.3
**Electrical burn**	14 (5.0%)	14 (6.3%)	0 (0.0%)	0.081
**Thermal burn**	267 (96.0%)	210 (95.0%)	57 (100.0%)	0.13
**Body surface area burned, %**	28.3 (20.0, 45.0)	25.0 (20.0, 40.0)	33.5 (20.0, 65.0)	0.005
**Full-thickness body surface area burned, %**	15.0 (5.0, 27.0)	11.5 (4.0, 24.3)	23.0 (14.0, 54.0)	<0.001
**Inhalation injury**	91 (32.7%)	56 (25.3%)	35 (61.4%)	<0.001
**ABSI**	8.0 (6.0, 10.0)	7.0 (6.0, 9.0)	10.0 (8.0, 12.0)	<0.001
**SAPS II**	29.0 (19.0, 41.0)	25.0 (16.8, 35.3)	47.0 (35.0, 63.0)	<0.001
**SOFA**	2.0 (0.0, 6.0)	1.0 (0.0, 4.0)	7.0 (3.0, 10.3)	<0.001
**Volume expansion with crystalloid fluids, ml/kg/%**	4.0 (2.4, 5.1)	3.9 (2.2, 5.0)	4.9 (3.2, 6.6)	0.017
**Vasopressors administration at admission**	107 (38.8%)	63 (28.8%)	44 (77.2%)	<0.001
**Length of vasopressors infusion, d**	1.0 (0.0, 3.0)	0.0 (0.0, 2.0)	2.0 (1.0, 12.0)	<0.001
**Mechanical ventilation at admission**	172 (61.9%)	125 (56.6%)	47 (82.5%)	<0.001
**Duration of mechanical ventilation, d**	3.0 (0.0, 30.8)	3.0 (0.0, 33.0)	4.0 (1.0, 21.0)	0.3
**Initial AST level, x ULN**	0.9 (0.7, 1.4)	0.9 (0.7, 1.4)	0.9 (0.8, 1.6)	0.7
**Initial ALT level, x ULN**	0.7 (0.5, 1.1)	0.7 (0.5, 1.0)	0.7 (0.5, 1.3)	0.3
**Initial GGT level, x ULN**	0.6 (0.4, 1.4)	0.6 (0.4, 1.3)	0.6 (0.4, 1.9)	0.2
**Initial ALP level, x ULN**	0.6 (0.5, 0.7)	0.6 (0.5, 0.7)	0.6 (0.5, 0.9)	0.003
**Initial BILT level, x ULN**	0.7 (0.4, 1.1)	0.6 (0.4, 1.0)	1.0 (0.6, 1.3)	<0.001
**Initial prothrombin ratio, %**	79.0 (64.0, 89.0)	81.0 (71.0, 90.0)	55.5 (41.0, 83.5)	<0.001
**Initial serum creatinine level, µmol/L**	72.0 (57.7, 94.0)	68.8 (56.9, 85.5)	101.0 (69.0, 120.0)	<0.001
**Enteral nutrition**	187 (67.3%)	150 (67.9%)	37 (64.9%)	0.7
**Parenteral nutrition**	18 (6.5%)	10 (4.5%)	8 (14.0%)	0.015
**Acute kidney injury**	95 (34.2%)	47 (21.3%)	48 (84.2%)	<0.001
**Renal replacement therapy**	39 (14.0%)	14 (6.3%)	25 (43.9%)	<0.001
**Acute respiratory distress syndrome**	65 (23.4%)	32 (14.5%)	33 (57.9%)	<0.001
**Septic shock**	76 (27.3%)	48 (21.7%)	28 (49.1%)	<0.001
**Total ketamine exposure, mg**	41.8 (0.0, 635.8)	50.0 (0.0, 556.9)	10.0 (0.0, 2,540.0)	0.6
**Ketamine dose distribution, mg**				0.031
[-Inf,0]	106 (38.1%)	79 (35.7%)	27 (47.4%)	
(0,1,000]	105 (37.8%)	93 (42.1%)	12 (21.1%)	
(1,000,10,000]	33 (11.9%)	25 (11.3%)	8 (14.0%)	
(10,000, Inf]	34 (12.2%)	24 (10.9%)	10 (17.5%)	
**Length of ketamine infusion, d**	1.0 (0.0, 6.0)	1.0 (0.0, 6.0)	1.0 (0.0, 4.0)	0.13

**Time to ketamine exposure> 10,000 mg**	6.0 (5.0, 8.8)	6.0 (6.0, 11.0)	5.0 (3.5, 6.0)	0.045
**Number of patients without ketamine infusion**	107 (38.5%)	80 (36.2%)	27 (47.4%)	0.12
**Total midazolam exposure, mg**	5.4 (0.0, 457.3)	5.0 (0.0, 419.2)	55.4 (0.0, 532.2)	0.7
**Length of midazolam infusion, d**	1.0 (0.0, 4.0)	1.0 (0.0, 4.0)	1.0 (0.0, 4.0)	0.9
**Number of patients without midazolam infusion**	121 (43.5%)	96 (43.4%)	25 (43.9%)	>0.9
**Total sufentanil exposure, µg**	169.9 (0.0, 2,527.8)	159.3 (0.0, 2,610.3)	269.3 (0.0, 1,421.5)	0.8
**Length of sufentanil infusion, d**	3.0 (0.0, 14.0)	3.0 (0.0, 16.0)	2.0 (0.0, 8.0)	0.3
**Number of patients without sufentanil infusion**	77 (27.7%)	61 (27.6%)	16 (28.1%)	>0.9
**Length of stay in the ICU**	29.5 (14.3, 48.0)	32.0 (19.0, 53.0)	10.0 (1.0, 31.0)	<0.001
Cholestasis was serum ALP ≥ 1.5 x ULN with GGT ≥ 3 x ULN; cholestatic liver injury was serum ALP ≥ 1.5 x ULN with GGT ≥ 3 x ULN and BILT > 1 x ULN; DILI cholestasis was serum ALP level ≥ 2 x ULN and serum GGT level ≥ 1 x ULN; Grade 3 or higher ALP elevation was serum ALP ≥ 5 x ULN. The SAPS II ranges from 0 to 163, with higher scores indicating greater severity of illness. The ABSI ranges from 0 to 18, with higher scores indicating a greater probability of death after burn injury. The SOFA ranges from 0 to 24 with higher scores indicate more severe organ failure. ABSI: abbreviated burn severity index; ALP: alkaline phosphatase; ALT: alanine aminotransferase ; AST: aspartate aminotransferase; BILT: total bilirubin; DILI: Drug induced liver injury; GGT: gamma-glutamyl transferase; Inf= Infinite; SAPS II: simplified acute physiology score II; SOFA: Sequential Organ Failure Assessment score; ULN : upper limit of normal				
1 n (%); Median (IQR)				
2 Pearson's Chi-squared test; Wilcoxon rank sum test; Fisher's exact test				

Table S3: Characteristics of ARDS patients by time period				
**Characteristic**	**Overall**, N = 65[1]	**Before ketamine reduction**, n = 34[1]	**After ketamine reduction**, n = 31[1]	p-value[2]
**Cholestatic liver injury**	21 (32.3%)	16 (47.1%)	5 (16.1%)	0.008
**Grade = 3 ALP elevation**	15 (23.1%)	12 (35.3%)	3 (9.7%)	0.014
**DILI cholestasis**	37 (56.9%)	21 (61.8%)	16 (51.6%)	0.409
**Cholestasis**	49 (75.4%)	27 (79.4%)	22 (71.0%)	0.430
**Male sex**	37 (56.9%)	19 (55.9%)	18 (58.1%)	0.859
**Age, years**	55.3 (39.3-68.1)	57.2 (44.7-68.9)	51.3 (34.9-63.3)	0.163
**Body mass index, Kg/m2**	26.1 (22.5-29.4)	26.7 (22.9-29.3)	26.1 (22.5-29.0)	0.622
**Electrical burn**	1 (1.5%)	0 (0.0%)	1 (3.2%)	0.477
**Thermal burn**	64 (98.5%)	34 (100.0%)	30 (96.8%)	0.477
**Body surface area burned, %**	40.0 (25.0-60.0)	39.0 (21.2-59.5)	45.0 (30.0-63.0)	0.747
**Full-thickness body surface area burned, %**	23.0 (12.0-50.0)	24.5 (13.2-40.8)	21.0 (10.8-52.0)	0.833
**Inhalation injury**	45 (69.2%)	25 (73.5%)	20 (64.5%)	0.432
**ABSI**	10.0 (8.0-12.0)	10.0 (8.0-11.8)	10.0 (7.5-12.0)	0.963
**SOFA**	8.0 (4.0-9.0)	7.5 (5.0-9.0)	8.0 (3.8-9.2)	0.775
**Volume expansion with crystalloid fluids, ml/kg/%**	4.8 (3.9-6.7)	5.1 (3.9-6.4)	4.4 (3.9-7.3)	0.727
**Vasopressors administration at admission**	46 (71.9%)	27 (79.4%)	19 (63.3%)	0.153
**Length of vasopressors infusion, d**	4.0 (1.0-13.0)	7.5 (1.0-17.5)	3.0 (1.0-7.5)	0.101
**Mechanical ventilation at admission**	63 (96.9%)	32 (94.1%)	31 (100.0%)	0.493
**Duration of mechanical ventilation, d**	24.0 (7.0-50.0)	21.5 (4.8-36.8)	27.0 (10.5-62.0)	0.438
**Initial AST level, x ULN**	1.1 (0.7-1.7)	1.1 (0.8-1.2)	1.5 (0.5-2.1)	0.689
**Initial ALT level, x ULN**	0.7 (0.5-1.1)	0.7 (0.5-1.1)	0.7 (0.6-1.0)	0.856
**Initial GGT level, x ULN**	0.6 (0.3-1.4)	0.5 (0.4-1.7)	0.8 (0.3-1.4)	0.908
**Initial ALP level, x ULN**	0.6 (0.5-0.8)	0.6 (0.5-0.9)	0.6 (0.5-0.8)	0.220
**Initial BILT level, x ULN**	0.7 (0.4-1.2)	0.8 (0.4-1.3)	0.7 (0.4-1.0)	0.270
**Initial prothrombin ratio, %**	67.0 (51.0-82.0)	64.0 (50.0-82.0)	69.5 (58.2-81.0)	0.638
**Initial serum creatinine level, µmol/L**	88.2 (67.3-118.0)	91.6 (67.3-118.9)	85.3 (67.5-115.1)	0.692
**Enteral nutrition**	52 (80.0%)	28 (82.4%)	24 (77.4%)	0.619
**Parenteral nutrition**	9 (13.8%)	7 (20.6%)	2 (6.5%)	0.153
**Acute kidney injury**	46 (70.8%)	26 (76.5%)	20 (64.5%)	0.290
**Renal replacement therapy**	27 (41.5%)	19 (55.9%)	8 (25.8%)	0.014
**Acute respiratory distress syndrome**	65 (100.0%)	34 (100.0%)	31 (100.0%)	
**Sepsis**	47 (72.3%)	26 (76.5%)	21 (67.7%)	0.432
**Number of sepsis**	2.0 (1.0-3.0)	2.0 (1.0-3.0)	2.0 (1.0-4.0)	0.350
**Septic shock**	37 (56.9%)	20 (58.8%)	17 (54.8%)	0.746
**Number of septic shock**	2.0 (1.0-3.0)	1.0 (1.0-2.0)	2.0 (1.0-3.2)	0.209

**Total ketamine exposure, mg**	75.0 (0.0-10,421.1)	8,623.8 (18.4-20,954.4)	0.0 (0.0-82.5)	<0.001
**Length of stay in the ICU**	32.0 (9.0-73.0)	28.5 (6.5-49.0)	52.0 (14.0-91.0)	0.166
**28-day mortality**	26 (40.0%)	17 (50.0%)	9 (29.0%)	0.085
**90-day mortality**	33 (50.8%)	23 (67.6%)	10 (32.3%)	0.004
Cholestasis was serum ALP ≥ 1.5 x ULN with GGT ≥ 3 x ULN; cholestatic liver injury was serum ALP ≥ 1.5 x ULN with GGT ≥ 3 x ULN and BILT > 1 x ULN; DILI cholestasis was serum ALP level ≥ 2 x ULN and serum GGT level ≥ 1 x ULN; Grade 3 or higher ALP elevation was serum ALP ≥ 5 x ULN. The SAPS II ranges from 0 to 163, with higher scores indicating greater severity of illness. The ABSI ranges from 0 to 18, with higher scores indicating a greater probability of death after burn injury. The SOFA ranges from 0 to 24 with higher scores indicate more severe organ failure. ABSI: abbreviated burn severity index; ALP: alkaline phosphatase; ALT: alanine aminotransferase ; AST: aspartate aminotransferase; BILT: total bilirubin; DILI: Drug induced liver injury; GGT: gamma-glutamyl transferase; Inf= Infinite; SAPS II: simplified acute physiology score II; SOFA: Sequential Organ Failure Assessment score; ULN : upper limit of normal				
1 n (%); Median (25%-75%)				
2 Pearson's Chi-squared test; Wilcoxon rank sum exact test; Wilcoxon rank sum test; Fisher's exact test				
