# Supplementary material for: Ketamine restriction correlates with reduced cholestatic liver injury and improved outcomes in critically ill patients with burn injury
Source: JHEP Rep. 2023 Nov 2;6(2):100950. doi: 10.1016/j.jhepr.2023.100950 (PMC10832380; doi:10.1016/j.jhepr.2023.100950)
Supplement: Multimedia component 2 — : [file mmc2.docx]

**JHEP Reports**

**CTAT methods**

Tables for a “Complete, Transparent, Accurate and Timely account” (CTAT) are now mandatory for all revised submissions. The aim is to enhance the reproducibility of methods.

- Only include the parts relevant to your study
- Refer to the CTAT in the main text as ‘Supplementary CTAT Table’
- Do not add subheadings
- Add as many rows as needed to include all information
- Only include one item per row

**If the CTAT form is not relevant to your study, please outline the reasons why:**

| **This study exclusively involved clinical work; there was no involvement of animal/biological research.** |
| --- |

- 1. **Antibodies**

| **Name** | **Citation** | **Supplier** | **Cat no.** | **Clone no.** |
| --- | --- | --- | --- | --- |
|  |  |  |  |  |

- 1. **Cell lines**

| **Name** | **Citation** | **Supplier** | **Cat no.** | **Passage no.** | **Authentication test method** |
| --- | --- | --- | --- | --- | --- |
|  |  |  |  |  |  |

- 1. **Organisms**

| **Name** | **Citation** | **Supplier** | **Strain** | **Sex** | **Age** | **Overall n number** |
| --- | --- | --- | --- | --- | --- | --- |
|  |  |  |  |  |  |  |

- 1. **Sequence based reagents**

| **Name** | **Sequence** | **Supplier** |
| --- | --- | --- |
|  |  |  |

- 1. **Biological samples**

| **Description** | **Source** | **Identifier** |
| --- | --- | --- |
|  |  |  |

- 1. **Deposited data**

| **Name of repository** | **Identifier** | **Link** |
| --- | --- | --- |
|  |  |  |

- 1. **Software**

| **Software name** | **Manufacturer** | **Version** |
| --- | --- | --- |
| **R** | **R Core Team (2023). _R: A Language and Environment for Statistical**  **Computing_. R Foundation for Statistical Computing, Vienna, Austria.**  **<https://www.R-project.org/>.** | **version 4.3.1 (2023-06-16): Beagle Scouts** |

- 1. **Other (*e.g*. drugs, proteins, vectors etc.)**

|  |  |  |
| --- | --- | --- |
|  |  |  |

- 1. **Please provide the details of the corresponding methods author for the manuscript:**

| Pr. Vincent Mallet, MD PhD, Assistance Publique-Hôpitaux de Paris, Hôpital Cochin, Hepatology service, 27 rue du Faubourg Saint Jacques, 75014 Paris, France E-mail, [vincent.mallet@aphp.fr](mailto:vincent.mallet@aphp.fr) Phone, + 33 1 58 41 30 01 Fax: + 33 1 58 41 30 14 |
| --- |

**2.0 Please confirm for randomised controlled trials all versions of the clinical protocol are included in the submission. These will be published online as supplementary information.**

|  |
| --- |
