# Supplementary material for: Ketamine restriction correlates with reduced cholestatic liver injury and improved outcomes in critically ill patients with burn injury
Source: JHEP Rep. 2023 Nov 2;6(2):100950. doi: 10.1016/j.jhepr.2023.100950 (PMC10832380; doi:10.1016/j.jhepr.2023.100950)
Supplement: Multimedia component 4 [file mmc4.pdf]

Research article JHEP|Reports

# Ketamine restriction correlates with reduced cholestatic liver injury and improved outcomes in critically ill patients with burn injury

Christian De Tymowski,[1,2,3,4,5] François Dépret,[1,4,6,7,8] Emmanuel Dudoignon,[1,4,8] Nabila Moreno,[9] Anne-Marie Zagdanski,[10] Kyann Hodjat,[4] Benjamin Deniau,[1,4,6,8] Alexandre Mebazaa,[1,4,6,8] Matthieu Legrand,[7,11] Vincent Mallet [1,4,12,*], for the Keta-Cov research group

*[1]Université Paris Cité, Paris, France; [2]Department of Anaesthesiology and Surgical Intensive Care Unit, Groupe Hospitalier Bichat Claude Bernard, DMU PARABOL, Assistance Publique–Hôpitaux de Paris, Paris, France; [3]Department of Anaesthesiology, Hôpital Louis Mourier, DMU PARABOL, Assistance Publique–Hôpitaux de Paris, Paris, France; [4]AP-HP.Nord, Groupe Hospitalier Saint Louis Lariboisière, DMU PARABOL, Département d'anesthésie réanimation et centre de traitement des brûlés, Paris, France; [5]Université Paris Cité, Centre de Recherche sur l'Inflammation, INSERM UMR 1149, CNRS ERL8252, Paris, France; [6]Institut National de la Santé et de la Recherche Médicale (INSERM), INSERM UMR-S 942 Mascot, Lariboisière Hospital, Paris, France; [7]INI-CRCT Network, Nancy, France; [8]FHU PROMICE, Paris, France; [9]AP-HP.Nord, Groupe Hospitalier Saint Louis Lariboisière, Laboratoire de Biochimie, Paris, France; [10]AP-HP.Nord, Groupe Hospitalier Saint Louis Lariboisière, Département de radiologie, Paris, France; [11]Department of Anesthesia and Peri-operative Care, Division of Critical Care Medicine, University of California, San Francisco, CA, USA; [12]Assistance Publique–Hôpitaux de Paris (AP-HP), Groupe Hospitalier Cochin Port Royal, DMU Cancérologie et spécialités médico-chirurgicales, Service de Maladie du Foie, Paris, France*

JHEP Reports **2024.** https://doi.org/10.1016/j.jhepr.2023.100950

**Background & Aims:** Ketamine-associated cholestatic liver injury is reported in patients with severe burn injury, but its association with patient outcome is unclear. We investigated the relationship between ketamine exposure, cholestatic liver injury, and outcome of critically ill patients with burn injury.
**Methods:** In a retrospective study, patients with severe burn injury were analysed across two periods: unrestricted ketamine prescription (ketamine-liberal) and capped ketamine dosage (ketamine-restricted). The primary endpoint was cholestatic liver injury, and the secondary endpoint was 3-month mortality. Binary logistic regression models and the revised electronic causality assessment method were used to measure the strength of associations and causality assessment, respectively.
**Results:** Of 279 patients (median age 51 [IQR 31–67] years; 63.1% men; burned surface area 28.5%, IQR 20–45%), 155 (56%) were in the ketamine-liberal group, and 124 (44%) were in the ketamine-restricted group, with comparable clinical characteristics, except for ketamine exposure (median doses 265.0 [IQR 0–8,021] mg and 20 [IQR 0–105] mg, respectively; $p$ <0.001). A dose- and time-dependent relationship was observed between ketamine exposure and cholestatic liver injury. Ketamine restriction was associated with a reduced risk of cholestatic liver injury (adjusted odds ratio 0.16, 95% CI 0.04–0.50; $p$ = 0.003) and with a higher probability of 3-month survival ($p$ = 0.035). The revised electronic causality assessment method indicated that ketamine was probably and possibly the cause of cholestatic liver injury for 14 and 10 patients, respectively. Cholangitis was not observed in the ketamine-restricted group. In propensity-matched patients, the risk of 3-month mortality was higher (adjusted odds ratio 9.92, 95% CI 2.76–39.05; $p$ = 0.001) in patients with cholestatic liver injury and ketamine exposure ≥10,000 mg. Other sedative drugs were not associated with liver and patient outcome.
**Conclusions:** In this cohort, ketamine restriction was associated with less cholestatic liver injury and reduced 3-month mortality.
**Impact and implications:** In a cohort of 279 critically ill patients with burn injury, ketamine was associated with a risk of liver bile duct toxicity. The risk was found to be dependent on both the dosage and duration of ketamine use. A restriction policy of ketamine prescription was associated with a risk reduction of liver injury and 3-month mortality. These findings have implications for the analgesia and sedation of critically ill patients with ketamine, with higher doses raising safety concerns.
© 2023 The Author(s). Published by Elsevier B.V. on behalf of European Association for the Study of the Liver (EASL). This is an open access article under the CC BY license (http://creativecommons.org/licenses/by/4.0/).

## Introduction

Ketamine is an intravenous hypnotic agent used in critically ill patients for rapid sequence induction[1]; for the management of acute and chronic pain,[2,3] including patients with severe burn injury[4]; and for maintenance sedation of patients with acute respiratory distress syndrome.[5]

Keywords: Ketamine; Cholestatic liver injury; Drug-induced liver injury; Drug toxicity; Mortality; Burn injury.
*Received 2 September 2023; accepted 29 September 2023; available online 2 November 2023*
* Corresponding author. Address: Assistance Publique–Hôpitaux de Paris, Hôpital Cochin, Hepatology Service, 27 rue du Faubourg Saint Jacques, 75014 Paris, France. Tel.: +33-1-58-41-30-01; Fax: +33-1-58-41-30-14.
*E-mail address:* vincent.mallet@aphp.fr (V. Mallet).

ELSEVIER
EASL The Home of Hepatology

Prolonged ketamine use has been associated with organ injuries, including ulcerative cystitis[6] and hepatic toxicities, such as cholestatic liver injury, cholangitis, and sclerosing cholangitis.[7] In 2017 and 2018, two pharmacovigilance alerts were released by the French National Agency for Medicines and Health Products Safety (ANSM): one on severe liver injuries following prolonged ketamine administration in patients with burn injury[8,9] and one on severe cholestatic liver injury, uro-nephrological injuries (including acute kidney injury), and endocrinological disturbances in patients or street users with prolonged/chronic ketamine exposure.[8,9]

We previously reported a ~10% rate of cholestatic liver injury in a 2012–2015 retrospective cohort of patients with severe burn injury.[10] Following the ANSM alert, we modified our ketamine prescription policy, and we observed a reduction in the incidence of cholestatic liver injury.[11] These observations suggested a dose-dependent drug-induced cholestatic liver injury. We therefore

**Table 1. Characteristics of patients by time period.**

| Characteristic | Overall, N = 279 (100%)* | Ketamine period | | p value† |
|---|---|---|---|---|
| | | Before reduction, n = 155 (56%)* | After reduction, n = 124 (44%)* | |
| Cholestatic liver injury | 34 (12.2) | 27 (17.4) | 7 (5.6) | 0.003 |
| Grade ≥3 ALP elevation | 25 (9.0) | 22 (14.2) | 3 (2.4) | <0.001 |
| Cholestasis | 175 (62.7) | 97 (62.6) | 78 (62.9) | 0.956 |
| DILI cholestasis | 106 (38.0) | 57 (36.8) | 49 (39.5) | 0.639 |
| Male sex | 176 (63.1) | 98 (63.2) | 78 (62.9) | 0.956 |
| Age (years) | 50.7 (31.4–67.3) | 49.1 (31.4–67.7) | 51.6 (31.3–67.1) | 0.860 |
| BMI (kg/m$^2$) | 25.1 (22.9–28.7) | 25.1 (22.8–28.7) | 25.1 (22.9–28.7) | 0.995 |
| Thermal burn | 268 (96.1) | 147 (94.8) | 121 (97.6) | 0.356 |
| Electrical burn | 14 (5.0) | 10 (6.5) | 4 (3.2) | 0.220 |
| Body surface area burned (%) | 28.5 (20.0–45.0) | 25.0 (20.0–45.0) | 30.0 (20.0–45.0) | 0.635 |
| Full-thickness body surface area burned (%) | 15.0 (5.0–27.8) | 14.0 (4.5–26.0) | 15.0 (6.0–28.5) | 0.219 |
| Inhalation injury | 91 (32.6) | 58 (37.4) | 33 (26.6) | 0.056 |
| ABSI | 8.0 (6.0–10.0) | 8.0 (6.0–10.0) | 8.0 (6.0–9.3) | 0.669 |
| SAPS II | 29.0 (19.0–41.0) | 33.0 (19.0–45.0) | 26.0 (18.5–37.0) | 0.066 |
| SOFA | 2.0 (0.0–6.0) | 3.0 (0.0–6.0) | 2.0 (0.0–6.0) | 0.981 |
| Volume expansion with crystalloid fluids (ml/kg/%) | 4.0 (2.4–5.3) | 4.0 (2.6–5.5) | 4.0 (2.3–5.0) | 0.602 |
| Vasopressors administration at admission | 108 (39.0) | 65 (41.9) | 43 (35.2) | 0.257 |
| Length of vasopressors infusion (days) | 1.0 (0.0–3.8) | 1.0 (0.0–4.0) | 1.0 (0.0–3.0) | 0.687 |
| Mechanical ventilation at admission | 173 (62.0) | 99 (63.9) | 74 (59.7) | 0.473 |
| Duration of mechanical ventilation (days) | 3.0 (0.0–31.0) | 3.0 (0.0–26.5) | 3.0 (0.0–34.3) | 0.483 |
| Initial AST level ( × ULN) | 0.9 (0.7–1.4) | 0.9 (0.7–1.2) | 1.0 (0.8–1.7) | 0.162 |
| Initial ALT level ( × ULN) | 0.7 (0.5–1.1) | 0.6 (0.5–1.1) | 0.7 (0.5–1.1) | 0.399 |
| Initial GGT level ( × ULN) | 0.6 (0.4–1.3) | 0.6 (0.4–1.5) | 0.6 (0.3–1.1) | 0.694 |
| Initial ALP level ( × ULN) | 0.6 (0.5–0.7) | 0.6 (0.5–0.7) | 0.6 (0.5–0.7) | 0.797 |
| Initial TBIL level ( × ULN) | 0.7 (0.4–1.1) | 0.7 (0.5–1.2) | 0.7 (0.4–1.1) | 0.880 |
| Initial prothrombin ratio (%) | 79.0 (64.0–89.0) | 79.0 (64.0–89.0) | 79.0 (65.0–88.5) | 0.998 |
| Initial serum creatinine level (µmol/L) | 71.5 (57.8–93.8) | 72.0 (57.1–94.5) | 71.0 (58.8–90.6) | 0.844 |
| Enteral nutrition | 188 (67.4) | 102 (65.8) | 86 (69.4) | 0.530 |
| Parenteral nutrition | 18 (6.5) | 12 (7.7) | 6 (4.8) | 0.327 |
| Acute kidney injury | 96 (34.4) | 57 (36.8) | 39 (31.5) | 0.352 |
| Renal replacement therapy | 39 (14.0) | 27 (17.4) | 12 (9.7) | 0.064 |
| Acute respiratory distress syndrome | 65 (23.3) | 34 (21.9) | 31 (25.0) | 0.547 |
| Septic shock | 76 (27.2) | 39 (25.2) | 37 (29.8) | 0.383 |
| Total ketamine exposure (mg) | 43.5 (0.0–624.1) | 265.0 (0.0–8,020.6) | 20.0 (0.0–105.0) | <0.001 |
| Length of ketamine infusion (days) | 1.0 (0.0–6.0) | 3.0 (0.0–9.0) | 1.0 (0.0–3.0) | <0.001 |
| Time to ketamine exposure >1,000 mg (days) | 2.0 (1.0–2.0) | 2.0 (1.0–2.0) | 50.0 (50.0–50.0) | 0.079 |
| Time to ketamine exposure >10,000 mg (days) | 6.0 (5.0–8.8) | 6.0 (5.0–8.8) | – | |
| Number of patients without ketamine infusion | 107 (38.4) | 52 (33.5) | 55 (44.4) | 0.065 |
| Total midazolam exposure (mg) | 5.8 (0.0–461.3) | 2.1 (0.0–336.1) | 26.5 (0.0–928.6) | 0.090 |
| Length of midazolam infusion (days) | 1.0 (0.0–4.0) | 1.0 (0.0–4.0) | 1.0 (0.0–7.0) | 0.033 |
| Number of patients without midazolam infusion | 121 (43.4) | 71 (45.8) | 50 (40.3) | 0.358 |
| Total sufentanil exposure (µg) | 170.9 (0.0–2,592.9) | 242.5 (0.0–1,455.6) | 164.1 (19.2–3,570.0) | 0.121 |
| Length of sufentanil infusion (days) | 3.0 (0.0–14.0) | 3.0 (0.0–10.0) | 3.0 (1.0–19.3) | 0.036 |
| Number of patients without sufentanil infusion | 77 (27.6) | 48 (31.0) | 29 (23.4) | 0.159 |
| Length of stay in the ICU (days) | 30.0 (14.5–48.5) | 26.0 (13.0–45.0) | 32.0 (19.0–58.0) | 0.023 |
| 28-day mortality | 43 (15.5) | 28 (18.1) | 15 (12.2) | 0.179 |
| 90-day mortality | 57 (20.5) | 39 (25.2) | 18 (14.6) | 0.031 |

Cholestasis was serum ALP ≥1.5ULN with GGT ≥3 × ULN; cholestatic liver injury was serum ALP ≥1.5 × ULN with GGT ≥3 × ULN, and TBIL >1 × ULN; DILI cholestasis was serum ALP ≥2 × ULN and serum GGT ≥1 × ULN; and grade 3 or higher ALP elevation was serum ALP ≥5 × ULN. The SAPS II ranges from 0 to 163, with higher scores indicating greater severity of illness. The ABSI ranges from 0 to 18, with higher scores indicating a greater probability of death after burn injury. The SOFA ranges from 0 to 24, with higher scores indicating more severe organ failure.

ABSI, abbreviated burn severity index; ALP, alkaline phosphatase; ALT, alanine aminotransferase; AST, aspartate aminotransferase; TBIL, total bilirubin; DILI, drug-induced liver injury; GGT, gamma glutamyl transferase; ICU, intensive care unit; SAPS II, Simplified Acute Physiology Score II; SOFA, Sequential Organ Failure Assessment; ULN, upper limit of normal.

* Data are presented as n (%) or median (IQR).

† Pearson's Chi-squared test, the Wilcoxon rank sum test, or Fisher's exact test.

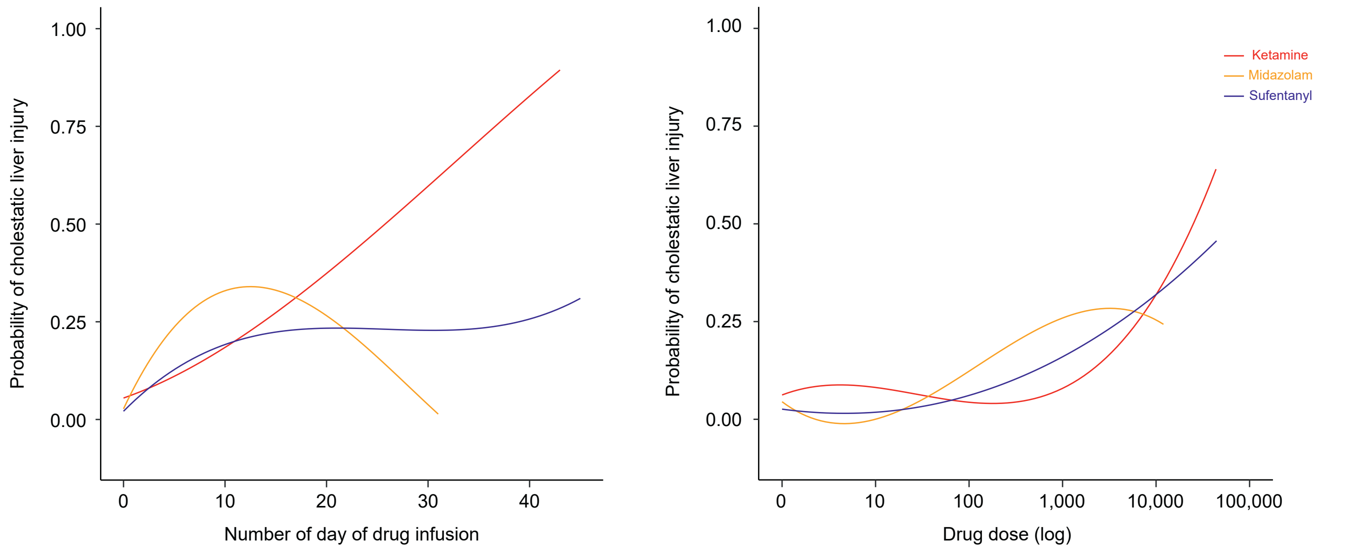


**Fig. 1. Probabilty of cholestatic liver injury according to the number of days of ketamine infusion and dose exposure.** Probabilities were computed using binary logistic regression according to the restrictive cubic spline method, using four knots.

explored the relationship between ketamine exposure, cholestatic liver injury, and 3-month mortality in our cohort.

## Patients and methods

### Setting

We conducted a retrospective, single-centre, cohort study in consecutive patients admitted to the Burn Intensive Care Unit (ICU) of Saint Louis Hospital (Assistance Publique–Hôpitaux de Paris, AP-HP, Paris, France) between December 2014 (start of electronic prescriptions) and June 2019. Patients were treated according to our local management protocols.[12] The study was approved by the local ethics committee (Comite de protection des personnes IV, St-Louis hospital; Institutional review board 00003835, protocol 2013/17NICB).

### Data sources

The data sources were the electronic records available (Diane, Bow Medical, France), the medical charts, the medical prescription database, and the biological data warehouse of the institution. Patient-level data check was performed for all patients by a senior investigator.

### Patients

Inclusion criteria were adult patients with at least one of the following: total burned surface area ≥20%, full thickness burned surface area ≥10%, and mechanical ventilation or vasopressor administration during the first 48 h after burn injury. Patients with the following criteria were excluded from the analysis: no liver function test performed during the burn ICU stay, and uncharted sedative and analgesic drugs prescription.

### Outcome measures

The primary outcome was cholestatic liver injury, which corresponded to the association of cholestasis (serum alkaline phosphatase [ALP] level ≥1.5 × upper limit of normal [ULN], with serum gamma glutamyl transferase [GGT] level ≥3 × ULN, and total serum bilirubin ≥1 × ULN).[10,13–15] We also considered other definitions for cholestatic liver injury, including serum ALP level ≥2 × ULN and serum GGT level ≥1 × ULN based on the drug-induced liver injury (DILI) definition[16] and grade 3 or higher serum ALP elevation (≥5 × ULN) based on the Common Terminology Criteria for Adverse Events (CTCAE).[17] The term *cholangitis* refers to prolonged inflammation and/or infection of the bile ducts. To assess the imputability of ketamine on cholestatic liver injury occurrence for each individual, we used the revised electronic causality assessment method (RECAM) (http://gihep.com/dili-recam/).[18,19] The secondary outcome was 3-month mortality.

### Exposures

We defined two time periods, according to ketamine prescription modalities[8,9]: a ketamine-liberal period, from December 2014 to end of March 2017, when ketamine prescription was 'liberally' used for maintenance sedation (≥1 mg/kg/h); and a ketamine-restricted period, from April 2017 to June 2019, when ketamine was used only as a second-line co-analgesic drug with a capped dose (<0.015 mg/kg/h) and not as a sedative agent. Other anaesthetic drug exposures, including total i.v. midazolam and sufentanil, were used as inner controls. Other exposures were patient demographics; burn characteristics, including inhalation injury; severity of illness scores, including the abbreviated burn severity index (ABSI; a score that ranges from 0 to 18, with higher scores indicating a greater probability of death after the burn injury), the Simplified Acute Physiology Score II (SAPS II; a score that ranges from 0 to 163, with higher scores indicating greater severity of illness),[20] and the Sequential Organ Failure Assessment (SOFA; a score that ranges from 0 to 24 with higher scores indicating more severe organ failure)[21]; initial crystalloid and norepinephrine administrations; critical care level (number of surgical procedures and parenteral nutrition); and organ failure, including acute kidney injury according to the Kidney Disease Improving Global Outcomes (KDIGO) criteria,[22] acute respiratory distress syndrome (ARDS) according to the Berlin definition,[23] and sepsis and septic shock according to the Sepsis-3 definition.[24]

### Statistical analysis

Associations were computed using backward stepwise binary logistic regression models. Variables with nominal two-tailed *p* values less than 0.1 were entered into the multivariate model, except for variables with obvious multicollinearity. Probabilities of cholestatic liver injury and 3-month mortality by ketamine dose were assessed by binary logistic regression according to the restrictive cubic spline method, using four knots. The knots were determined to have homogenous population distribution. As 38% of the patients did not receive any ketamine, the population could not be cut into quartiles, and we divided the population into four groups, namely, 38%, 38%, 12%, and 12% of the population. To address confounding by indication of ketamine and other source of bias arising from observational data, we estimated a full propensity score matching, without replacement, using the Matchit and Optmatch packages.[25,26] Propensity scores were estimated using logistic regression of the likelihood of total ketamine doses ≥1,000 mg, or not, on severity of illness and organ failures (see Fig. S1). We used the 1,000 mg threshold for the two ketamine exposure periods because the risk of cholestatic liver injury increased beyond this threshold (see Fig. 1). All statistical tests were based on two-tailed *p* values, with $p < 0.05$ considered to indicate statistical significance. Missing data were not imputed. All analyses were performed using R statistical software (R 4.2.2 GUI 1.79 Big Sur ARM build [8160]).

## Results

### Characteristics of patients

Of the 885 patients included in the study, 279 (median age 51 [IQR 31–67] years; 63.1% men) were eligible for analysis: 155 (56%) during the ketamine-liberal period and 124 (44%) during the ketamine-restricted period (see Fig. 2). The majority of patients (96.1%) were admitted after thermal burn injuries, with a median burned body surface area of 28.5%, and one-third (32.6%) had inhalation injury. Patient characteristics by period are outlined in Table 1, demonstrating no statistically significant differences between the two groups in initial severity scores (SAPS II, ABSI, and SOFA), initial resuscitation procedures, and organ failures during the ICU stay, including duration of vasopressor infusion and mechanical ventilation, acute kidney injury, renal replacement therapy, acute respiratory syndrome, sepsis, and septic shock. Liver tests at admission were also comparable between the two periods.

Median (IQR) ketamine doses during the ketamine-liberal and ketamine-restricted periods were 265.0 (0.0–8,020.6) and 20 (0–105) mg, respectively ($p < 0.001$). The total number of days with ketamine infusion was lower ($p < 0.001$) during the ketamine-restricted period. There was a trend towards higher doses of midazolam ($p = 0.090$), but not of sufentanil ($p = 0.12$), and longer exposures to midazolam ($p = 0.033$) and sufentanil ($p = 0.036$) in ketamine-restricted patients. The ketamine-liberal period was associated with a shorter ICU stay ($p = 0.023$) and higher 3-month mortality ($p = 0.031$).

During the study, 34 (12%) patients developed cholestatic liver injury. Being in the ketamine-restricted period was associated with fewer cholestatic liver injuries ($p = 0.003$), fewer grade ≥3 serum ALP elevations ($p < 0.001$), and lower serum GGT levels. However, the prevalence of cholestasis ($p = 0.956$) and drug-induced liver injury cholestasis (serum ALP ≥2 × ULN and GGT >1 × ULN; $p = 0.639$) remained similar between the two periods.

Fig. 3 displays the evolution of liver tests during the ICU stay by ketamine period, showing a progressive elevation of serum ALP, GGT, and total bilirubin in the ketamine-liberal period and not ($p < 0.001$) in the ketamine-restricted period. The evolution of liver tests by year is depicted in Fig. S2, indicating that, except for serum GGT, liver tests remained generally stable during the study period.

### Ketamine and risk of cholestatic liver injury

Cholestatic liver injury was associated with severity of illness; critical care level, including number of surgical procedures ($p < 0.001$) and parenteral nutrition; total sedative drugs doses ($p < 0.001$); ketamine-liberal period ($p = 0.003$); 3-month mortality ($p = 0.001$), and not 28-day mortality ($p = 0.2$). Other characteristics are detailed in Table 2. In multivariate analysis (Table 3), the ketamine-restricted period was associated with a lower risk of cholestatic liver injury (adjusted odds ratio [AOR] 0.11, 95% CI 0.02–0.44; $p = 0.003$). Other associated factors were inhalation injury ($p = 0.008$), number of surgical procedures ($p = 0.018$), parenteral nutrition ($p = 0.039$), renal replacement therapy ($p = 0.002$), and sepsis ($p = 0.017$). ARDS was not an independent

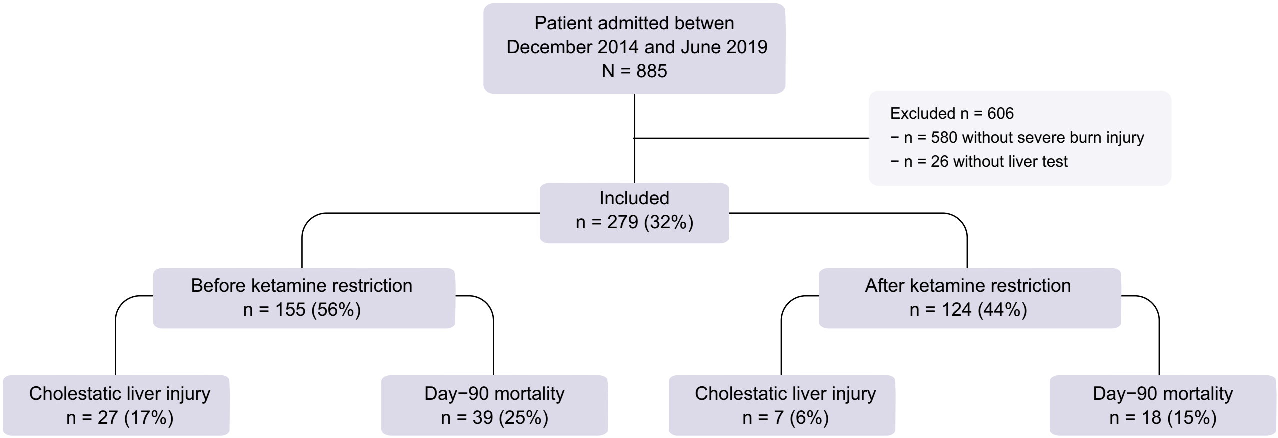


**Fig. 2. Study flow chart.** Cholestatic liver injury corresponded to the association of cholestasis (serum ALP level ≥1.5 × ULN, with serum GGT level ≥3 × ULN) and hyperbilirubinaemia (total serum bilirubin ≥1 × ULN). ALP, alkaline phosphatase; GGT, gamma glutamyl transferase; ULN, upper limit of normal.

**Table 2. Characteristics of patients by cholestatic liver injury.**

| Characteristic | Overall, N = 279 (100%)* | No cholestatic liver injury, n = 245 (88%)* | Cholestatic liver injury, n = 34 (12%)* | *p* value† |
|---|---|---|---|---|
| Male sex | 176 (63.1) | 154 (62.9) | 22 (64.7) | 0.834 |
| Age (years) | 50.7 (31.4–67.3) | 50.6 (31.0–68.1) | 50.8 (36.0–63.2) | 0.994 |
| BMI (kg/m$^2$) | 25.1 (22.9–28.7) | 24.8 (22.9–28.5) | 26.1 (23.2–29.3) | 0.276 |
| Electrical burn | 14 (5.0) | 14 (5.7) | 0 (0.0) | 0.231 |
| Thermal burn | 268 (96.1) | 235 (95.9) | 33 (97.1) | >0.999 |
| Body surface area burned (%) | 28.5 (20.0–45.0) | 25.0 (20.0–40.0) | 42.5 (30.0–61.5) | 0.002 |
| Full-thickness body surface area burned (%) | 15.0 (5.0–27.8) | 13.5 (4.0–25.0) | 30.0 (15.0–52.0) | <0.001 |
| Inhalation injury | 91 (32.6) | 67 (27.3) | 24 (70.6) | <0.001 |
| ABSI | 8.0 (6.0–10.0) | 8.0 (6.0–9.0) | 10.0 (7.0–11.0) | <0.001 |
| SAPS II | 29.0 (19.0–41.0) | 28.0 (18.0–40.0) | 38.0 (31.0–46.0) | <0.001 |
| SOFA | 2.0 (0.0–6.0) | 2.0 (0.0–5.0) | 7.0 (3.8–9.3) | <0.001 |
| Volume expansion with crystalloid fluids (ml/kg/%) | 4.0 (2.4–5.3) | 3.9 (2.2–5.0) | 4.9 (4.0–6.0) | 0.032 |
| Vasopressors administration at admission | 108 (39.0) | 85 (35.0) | 23 (67.6) | <0.001 |
| Length of vasopressors infusion (days) | 1.0 (0.0–3.8) | 1.0 (0.0–2.0) | 9.0 (2.0–17.8) | <0.001 |
| Mechanical ventilation at admission | 173 (62.0) | 140 (57.1) | 33 (97.1) | <0.001 |
| Duration of mechanical ventilation (days) | 3.0 (0.0–31.0) | 2.0 (0.0–26.0) | 36.0 (18.5–67.5) | <0.001 |
| Initial AST level ( × ULN) | 0.9 (0.7–1.4) | 0.9 (0.7–1.4) | 1.2 (0.8–1.6) | 0.615 |
| Initial ALT level ( × ULN) | 0.7 (0.5–1.1) | 0.7 (0.5–1.0) | 0.8 (0.5–1.2) | 0.272 |
| Initial GGT level ( × ULN) | 0.6 (0.4–1.3) | 0.6 (0.3–1.2) | 0.6 (0.4–1.9) | 0.097 |
| Initial ALP level ( × ULN) | 0.6 (0.5–0.7) | 0.6 (0.5–0.7) | 0.6 (0.5–0.8) | 0.150 |
| Initial TBIL level ( × ULN) | 0.7 (0.4–1.1) | 0.7 (0.4–1.0) | 0.9 (0.4–1.3) | 0.277 |
| Initial prothrombin ratio (%) | 79.0 (64.0–89.0) | 80.0 (66.0–89.0) | 69.0 (46.5–83.0) | 0.013 |
| Initial serum creatinine level (μmol/L) | 71.5 (57.8–93.8) | 70.0 (57.1–89.2) | 86.0 (68.1–118.0) | 0.028 |
| Enteral nutrition | 188 (67.4) | 158 (64.5) | 30 (88.2) | 0.006 |
| Parenteral nutrition | 18 (6.5) | 8 (3.3) | 10 (29.4) | <0.001 |
| Acute kidney injury | 96 (34.4) | 70 (28.6) | 26 (76.5) | <0.001 |
| Renal replacement therapy | 39 (14.0) | 19 (7.8) | 20 (58.8) | <0.001 |
| Acute respiratory distress syndrome | 65 (23.3) | 44 (18.0) | 21 (61.8) | <0.001 |
| Septic shock | 76 (27.2) | 53 (21.6) | 23 (67.6) | <0.001 |
| Ketamine dose reduction period | | | | 0.003 |
| Before reduction | 155 (55.6) | 128 (52.2) | 27 (79.4) | |
| After reduction | 124 (44.4) | 117 (47.8) | 7 (20.6) | |
| Total ketamine exposure (mg) | 43.5 (0.0–624.1) | 30.0 (0.0–354.0) | 9,936.2 (70.0–19,547.2) | <0.001 |
| Ketamine dose exposure distribution (mg) | | | | <0.001 |
| [-Inf, 0] | 106 (38.0) | 100 (40.8) | 6 (17.6) | |
| (0, 1,000] | 106 (38.0) | 100 (40.8) | 6 (17.6) | |
| (1,000, 10,000] | 33 (11.8) | 28 (11.4) | 5 (14.7) | |
| (10,000, Inf] | 34 (12.2) | 17 (6.9) | 17 (50.0) | |
| Length of ketamine infusion (days) | 1.0 (0.0–6.0) | 1.0 (0.0–4.0) | 8.5 (2.3–20.8) | <0.001 |
| Time to ketamine exposure >1,000 mg | 2.0 (1.0–2.0) | 2.0 (1.0–2.5) | 2.0 (1.0–2.0) | 0.639 |
| Time to ketamine exposure >10,000 mg | 6.0 (5.0–8.8) | 6.0 (4.5–7.0) | 6.0 (5.5–13.0) | 0.285 |
| Number of patients without ketamine infusion | 107 (38.4) | 101 (41.2) | 6 (17.6) | 0.008 |
| Total midazolam exposure (mg) | 5.8 (0.0–461.3) | 2.0 (0.0–327.9) | 539.6 (234.2–1,526.1) | <0.001 |
| Length of midazolam infusion (days) | 1.0 (0.0–4.0) | 1.0 (0.0–4.0) | 6.0 (3.0–10.0) | <0.001 |
| Number of patients without midazolam infusion | 121 (43.4) | 116 (47.3) | 5 (14.7) | <0.001 |
| Total sufentanil exposure (μg) | 170.9 (0.0–2,592.9) | 90.0 (0.0–1,735.8) | 3,247.3 (685.2–4,967.1) | <0.001 |
| Length of sufentanil infusion (days) | 3.0 (0.0–14.0) | 2.0 (0.0–12.0) | 12.5 (6.3–27.5) | <0.001 |
| Number of patients without sufentanil infusion | 77 (27.6) | 75 (30.6) | 2 (5.9) | 0.003 |
| Length of stay in the ICU (days) | 30.0 (14.5–48.5) | 28.0 (13.0–46.0) | 55.0 (27.3–91.0) | <0.001 |
| 28-day mortality | 43 (15.5) | 35 (14.3) | 8 (23.5) | 0.165 |
| 90-day mortality | 57 (20.5) | 43 (17.6) | 14 (41.2) | 0.001 |

Cholestatic liver injury was serum ALP ≥1.5 × ULN with GGT ≥3 × ULN and TBIL >1 × ULN. The SAPS II ranges from 0 to 163, with higher scores indicating greater severity of illness. The ABSI ranges from 0 to 18, with higher scores indicating a greater probability of death after burn injury. The SOFA ranges from 0 to 24, with higher scores indicating more severe organ failure.
ABSI, abbreviated burn severity index; ALP, alkaline phosphatase; ALT, alanine aminotransferase; AST, aspartate aminotransferase; TBIL, total bilirubin; GGT, gamma glutamyl transferase; ICU, intensive care unit; Inf, infinite; SAPS II, Simplified Acute Physiology Score II; SOFA, Sequential Organ Failure Assessment; ULN, upper limit of normal.
* Data are presented as n (%) or median (IQR).
† Pearson's Chi-squared test, the Wilcoxon rank sum test, or Fisher's exact test.

risk factor for cholestatic liver injury. In the subgroup of patients with ARDS (n = 65; 23.2%), cholestatic liver injury ($p$ = 0.008), acute kidney injury ($p$ = 0.014), and 3-month mortality ($p$ = 0.004) were electively associated with the ketamine-liberal period (see Table S1). There was a linear relationship between the duration of ketamine exposure and the risk of cholestatic liver injury (Fig. 1, left). The association between cholestatic liver injury and ketamine dose was not linear but increased with total ketamine doses ≥1,000 mg (Fig. 1, right). There was no relationship between total midazolam and sufentanil exposures and cholestatic liver injury. Total sufentanil and midazolam doses were not independent risk factors of cholestatic liver injury. Similar results were obtained with other definitions of cholestasis (not shown).

**Table 3. Adjusted probabilities of cholestatic liver injury after severe burn injury.**

| Risk factors | Adjusted odds ratio (95% CI) | *p* value |
|---|---|---|
| Ketamine-restricted period | 0.16 (0.04–0.50) | 0.003 |
| Inhalation injury | 4.33 (1.51–13.67) | 0.008 |
| Number of surgical procedures | 1.18 (1.04–1.37) | 0.018 |
| Parenteral nutrition | 4.29 (1.10–18.10) | 0.039 |
| Acute respiratory distress syndrome | 2.41 (0.81–7.29) | 0.113 |
| Renal replacement therapy | 5.53 (1.86–17.03) | 0.002 |
| Sepsis | 5.53 (1.49–26.91) | 0.017 |
| Observations | | 276 |
| $R^2$ Tjur | | 0.506 |

Risks were computed using backward stepwise binary logistic regression models adjusted for body surface area burned, full thickness body surface burned, severity of illness, intensity of critical care, and total sufentanil and midazolam dose. Cholestatic liver injury was serum ALP ≥1.5 × N with GGT ≥3 × N and TBIL >ULN.
ALP, alkaline phosphatase; TBIL, total bilirubin; GGT, gamma glutamyl transferase; N, normal; ULN, upper limit of normal.

### Causality assessment between ketamine and cholestatic liver injury

The medical records of all patients with cholestatic liver injury are documented in Table S1. A total of 19 (54%) patients with cholestatic liver injury progressed to overt cholangitis, characterised by grade 3 (≥5 N) ALP elevation (n = 16), unexplained prolonged cholestasis (n = 11), biliary sepsis (n = 3), and one case of multiple liver abscess. In addition, four patients had progressive bile duct strictures and dilatations. Magnetic resonance cholangiopancreatography during the ICU stay revealed biliary casts and figures of sclerosing cholangitis in one case (Fig. S3). All patients with cholangitis, except one, were in the ketamine-liberal group. The median total ketamine doses for patients with and without cholangitis were 17,219 and 50 mg, respectively. The single ketamine-restricted patient with common bile duct dilatation had chronic hepatitis C and was undergoing opioid substitutive therapy. Sepsis preceded cholestatic liver injury for 26 (74%) patients, with a median delay of 5 days. Only two patients experienced very early cholestatic liver injury after burn injury (1 day), and two patients without ketamine had cholestatic liver injury without any apparent risk factor. A *post hoc* RECAM analysis of all cholestatic liver injury cases indicated that ketamine was most likely the cause for 3 patients, probably the cause for 11, and possibly the cause for 10 cases. During the ketamine-liberal period, ketamine was unlikely the cause of cholestatic liver injury for five patients, three of whom did not receive ketamine. One patient received ketamine after the onset of cholestatic liver injury, and one patient's liver function temporarily improved while still receiving ketamine.

### Ketamine-associated cholestatic liver injury and 3-month mortality

In univariate analysis (Table S2), ketamine dose reduction period ($p$ = 0.031), ketamine dose exposure ≥1,000 mg ($p$ = 0.031), and cholestatic liver injury ($p$ = 0.001) were associated with 3-month mortality, along with severity of illness. Midazolam and sufentanil dose exposures were not associated with patient 3-month survival. The ketamine-restricted group had lower 3-month mortality (see Fig. 4; $p$ = 0.035 with the log-rank test) with an AOR of 0.35 (95% CI 0.15–0.79; $p$ = 0.014; Table 4). In a propensity-matched sample (Table 5), the risk of 3-month mortality was highest with cholestatic liver injury when total ketamine doses were ≥10,000 mg, with an AOR of 9.92 (95% CI 2.76–39.05; $p$ = 0.001). Cholestatic liver injury without ketamine doses ≥10,000 mg ($p$ = 0.894) and ketamine doses ≥10,000 mg without cholestatic liver injury ($p$ = 0.446) were not associated with patient outcome.

## Discussion

### Summary of the main results

We report a dose- and time-dependent relationship between ketamine and the risk of cholestatic liver injury in critically ill patients with burn injury. The risk increased for ketamine exposures ≥1,000 mg and was maximal for exposures ≥10,000 mg. By contrast, we did not observe any time- or dose-dependent relationship between cholestatic liver injury and midazolam or sufentanil, two drugs commonly given to critically ill patients for sedation and analgesia. A reduction in the prescription policy of ketamine reduced (by ~80%) the risk of cholestatic liver injury, and cholangitis, including severe forms of sclerosing cholangitis such as progressive sclerosing cholangitis, and was associated with lower (~60%) 3-month mortality. The causality assessment, including patient-level check, and propensity scores matching, suggested that ketamine contributed to cholestatic liver injury in this cohort.

### Added value of the study

This study is the first to report a connection between a reduction in ketamine dose exposure and improved liver and patient outcomes. Our findings are consistent with previous reports of ketamine-associated liver toxicities, seen in various patient populations such as those undergoing anaesthesia,[27] drug-abuse users,[7] patients with chronic pain,[28] patients with burn injury,[29] critically ill patients,[30] and those receiving ketamine for maintenance sedation during the COVID-19 pandemic.[31–33] The link between ketamine-associated cholestatic liver injury and 3-month mortality is supported by reports of liver-related deaths in patients with COVID-19 exposed to high ketamine doses,[31,34] emphasising the importance of considering hepatic dysfunction, including cholestatic liver injury, in critical care settings.[35,36] Our study's observation of a dose-dependent relationship between ketamine exposure and cholestatic liver injury is consistent with animal models[37] and previous reports of a dose–effect relationship between long-term ketamine infusion and rising total bilirubin levels.[33]

The study is also first to provide evidence of a time- and dose-dependent relationship between ketamine and cholestatic liver injury. In addition, it stands out as the first study to thoroughly assess causality for each event and use propensity score matching in the context of ketamine-induced cholestatic liver injury.

### Meaning of the study

In this cohort, we observed both a linear relationship and an exponential relationship between the duration of ketamine exposure and total ketamine drug exposure, and the occurrence of cholestatic liver injury. Notably, it took only 2 and 5 days to reach 1,000 and 10,000 mg of total ketamine drug exposure, respectively.[10] The risk of cholestasis, regardless of jaundice, was similar in patients under both ketamine-restricted and ketamine-liberal conditions, suggesting that biliary tract injury is common in patients with severe burn injury, regardless of ketamine use.[10]

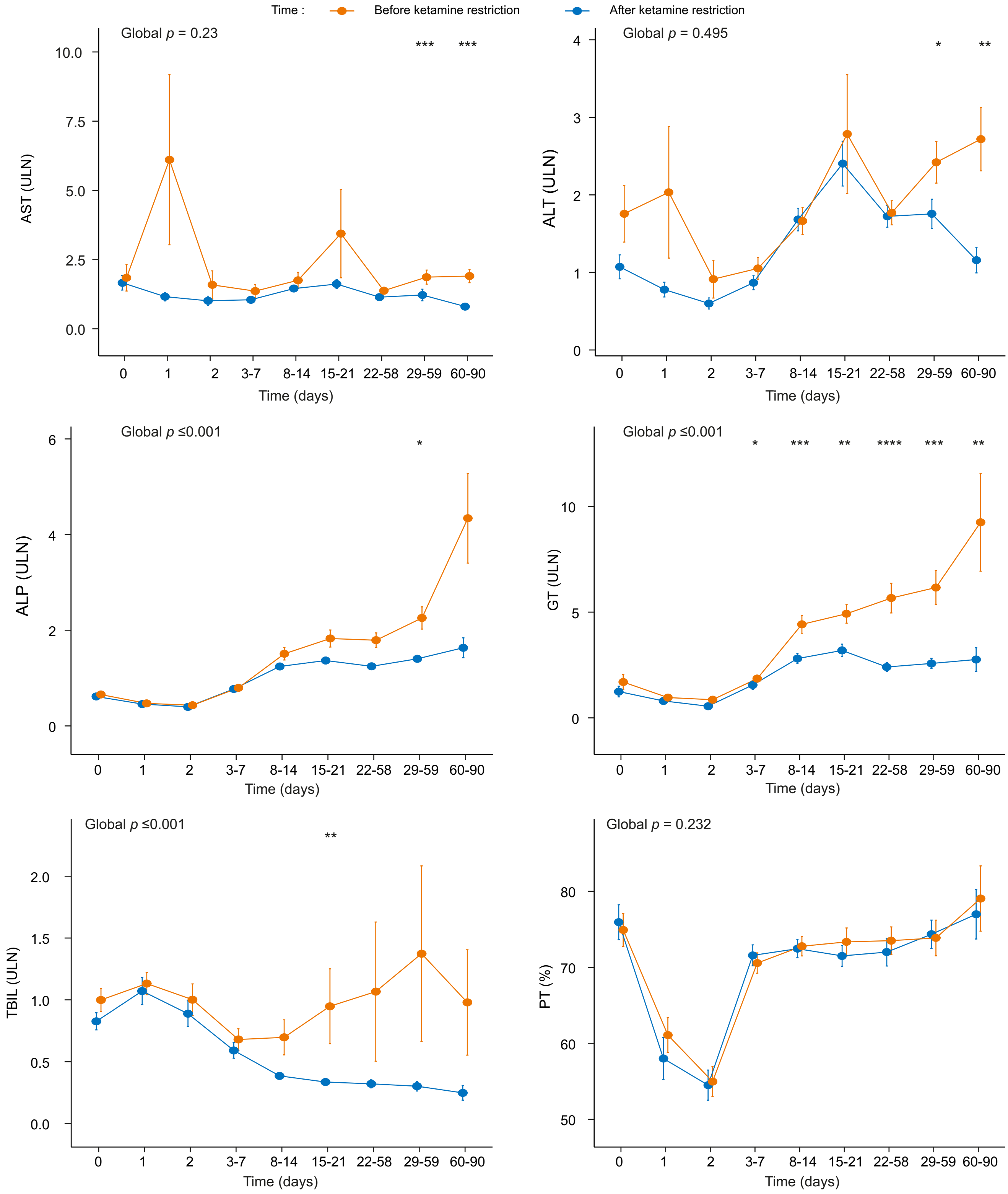


**Fig. 3. Liver tests evolution by ketamine period.** Global p values were computed using a mix model. The two-groups comparison at each time point were performed using the Mann–Whitney U test, (n.s., $p$ >0.05; *$p$ <0.05; **$p$ <0.01; ***$p$ <0.001; ****$p$ <0.0001). We defined two time periods, according to ketamine prescription modalities: a ketamine-liberal period, from December 2014 to end of March 2017, when ketamine prescription was 'liberally' used for maintenance sedation (≥ 1 mg/kg/h), and a ketamine-restricted period, from April 2017 to June 2019, when ketamine was only used as a second line co-analgesic drug with a capped dose (<0.015 mg/kg/h). ALP, alkaline phosphatase; ALT, alanine aminotransferase; AST, aspartate aminotransferase; PT, prothrombin time; TBIL, total bilirubin; ULN, upper limit of normal.

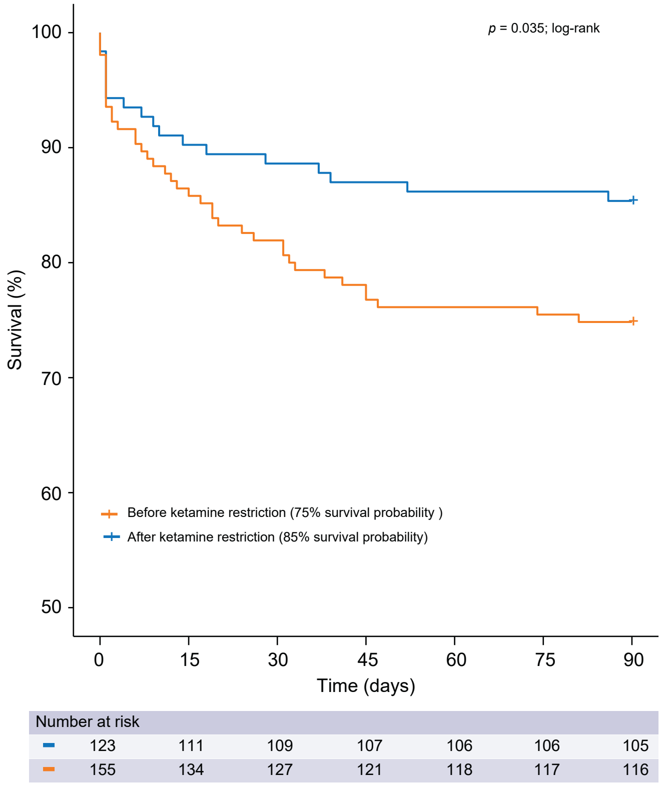


**Fig. 4. Probability of 3-month survival by ketamine restriction period.** We defined two time periods, according to ketamine prescription modalities: a ketamine-liberal period, from December 2014 to end of March 2017, when ketamine prescription was 'liberally' used for maintenance sedation (≥1 mg/kg/h), and a ketamine-restricted period, from April 2017 to June 2019, when ketamine was only used as a second line co-analgesic drug with a capped dose (<0.015 mg/kg/h).

**Table 4. Adjusted probabilities for 3-month mortality.**

| | 3-month mortality | |
|---|---|---|
| **Risk factors** | **Adjusted odds ratio (95% CI)** | ***p* value** |
| Age | 1.04 (1.02–1.06) | <0.001 |
| ABSI | 1.33 (1.13-1.58) | 0.001 |
| Acute kidney injury | 8.14 (3.47–20.72) | <0.001 |
| Acute respiratory distress syndrome | 3.45 (1.47–8.29) | 0.005 |
| Ketamine-restricted period | 0.35 (0.15–0.79) | 0.014 |
| Observations | | 278 |
| $R^2$ Tjur | | 0.409 |

Risks were computed using logistic regression models. The ABSI ranges from 0 to 18, with higher scores indicating a greater probability of death after the burn injury. ABSI, abbreviated burn severity index.

Burn-associated biliary injury likely involves a systemic inflammatory response, shock-induced bile duct ischaemia, sepsis, and beta-lactam.[38] There may be a modification of microsomal cytochrome P450 metabolism after burn injury, which could favour ketamine toxicity.[39]

Our findings suggest that ketamine acts as an additive factor of liver injury in patients with burn-associated biliary injury. Cholangitis, including progressive sclerosing cholangitis, did not occur in patients exposed to low doses of ketamine.

**Table 5. Adjusted probabilities for 3-month mortality in propensity-matched patients.**

| | 3-month mortality | |
|---|---|---|
| **Risk factors** | **Adjusted odds ratio (95% CI)** | ***p* value** |
| ABSI | 1.11 (0.97–1.26) | 0.122 |
| SAPS II | 1.04 (1.03–1.06) | <0.001 |
| Acute kidney injury | 0.88 (0.37–2.00) | 0.756 |
| Acute respiratory distress syndrome | 0.86 (0.33–2.18) | 0.751 |
| Cholestatic liver injury with ketamine ≥10,000 mg | 9.92 (2.76–39.05) | 0.001 |
| No cholestatic liver injury with ketamine ≥10,000 mg | 0.43 (0.02–2.64) | 0.446 |
| Cholestatic liver injury with ketamine <10,000 mg | 0.90 (0.14–3.78) | 0.894 |
| Total sufentanil (mg): (0, 50] | 1.77 (0.54–5.71) | 0.340 |
| Total sufentanil (mg): (50, 1,500] | 0.58 (0.17–1.86) | 0.367 |
| Total sufentanil (mg): (1,500, 5,000] | 0.42 (0.08–1.98) | 0.281 |
| Total sufentanil (mg): (5,000, Inf] | 0.23 (0.04–1.36) | 0.110 |
| Total midazolam (mg): (0, 500] | 1.97 (0.68–5.92) | 0.216 |
| Total midazolam (mg): (500, 1,500] | 2.95 (0.61–14.37) | 0.177 |
| Total midazolam (mg): (1,500, Inf] | 1.31 (0.21–8.08) | 0.768 |
| Observations | | 279 |

Patient were matched (full matching) on the probability of receiving ketamine dose >1,000 mg according to severity of illness (ABSI and SAPS II scores), and organ failure (acute kidney injury, acute respiratory syndrome, and septic shock). Risks were computed with multivariate logistic regression models. Cholestatic liver injury corresponded to an increase of ALP ≥1.5 × ULN with a concomitant increase of serum GGT ≥3 × ULN and TBIL >ULN. The SAPS II ranges from 0 to 163, with higher scores indicating greater severity of illness. The ABSI ranges from 0 to 18, with higher scores indicating a greater probability of death after the burn injury.
ABSI, abbreviated burn severity index; ALP, alkaline phosphatase; TBIL, total bilirubin; GGT, gamma glutamyl transferase; Inf, infinite; SAPS II, Simplified Acute Physiology Score II; ULN, upper limit of normal.

Ketamine is biotransformed in the liver with multiple metabolites. The most important pathway involves N-demethylation of ketamine to norketamine, a water-insoluble by-product, by cytochrome P450 (CYP 3A4) in the liver. Norketamine is then hydroxylated and conjugated to water-soluble compounds that are excreted in the urine.[40] Norketamine has been found in the bile and urine after fatal ketamine poisoning.[41] Ketamine-associated hepatobiliary injuries are thought to be the consequence of a direct effect of ketamine or toxic intermediates on the biliary epithelial cell.[42]

Although liver injury is associated with ICU patient mortality,[43] ketamine may also have favoured patients' mortality by increasing the risk of acute kidney injury.[44] The association between cholestatic liver injury and renal replacement therapy in our cohort was consistent with the idea of an accumulation of ketamine or toxic, hydrophobic by-products in the liver and the kidney.[42]

## Strengths and weaknesses of the study

One strength of our study is the before and after comparison of two different periods, which helped limit the risk of unobserved confounding. Patients were compatible between the two periods, and the overall liver tests remained unchanged during the study. Medical practice did not change during the two study periods, with the exception of ketamine prescriptions.[12] We also normalised the analyses on sufentanil and midazolam prescription, two drugs without known liver toxicity, to reduce the risk of unobserved confounding. A limitation could be the relatively small effective population, although the sample size was comparable with other reports on ketamine toxicity.[7,33] To address this limitation, we used a 'full matching' method with propensity

score matching, which has the advantage of retaining the whole population by assigning a weight to each patient. The generalisability of our findings may be constrained as they could apply solely to patients with severe burn injuries. It is noteworthy that ketamine toxicity has consistently been reported after chronic administration or misuse, and recent reports of liver-related deaths in patients with COVID-19 exposed to ketamine[31] suggest that our results may be transposable to other conditions. Ketamine may have been overlooked in studies reporting on critical care sclerosing cholangitis.[34]

## Conclusions

In a population at risk for liver injury, high doses of ketamine increased the risk of cholestatic liver injury, cholangitis, and mortality, and a ketamine prescription restriction policy improved patient outcome. Ketamine should be used with caution in critical care patients.[45] Liver test monitoring is mandatory for ketamine dose ≥1,000 mg. Ketamine should be considered as a potential culprit when investigating causes of cholangitis (or of abnormal liver tests) in critically ill patients.

## Abbreviations

ABSI, abbreviated burn severity index; ALP, alkaline phosphatase; ALT, alanine aminotransferase; ANSM, French National Agency for Medicines and Health Products Safety; AOR, adjusted odds ratio; ARDS, acute respiratory distress syndrome; AST, aspartate aminotransferase; CTCAE, Common Terminology Criteria for Adverse Events; DILI, drug-induced liver injury; GGT, gamma glutamyl transferase; ICU, intensive care unit; IQR, interquartile range; KDIGO, Kidney Disease Improving Global Outcomes; RECAM, revised electronic causality assessment method; SAPS II, Simplified Acute Physiology Score II; SOFA, Sequential Organ Failure Assessment; ULN, upper limit of normal.

## Financial support

The study did not receive any private or public funding.

## Conflicts of interest

The authors of this study declare that they do not have any conflict of interest Please refer to the accompanying ICMJE disclosure forms for further details.

## Authors' contributions

Conception of the study, analysis and interpretation of the data, and draft of the manuscript: VM, CDT
Data collection: CDT, ED, KH, AMZ, NM,
Writing – review and editing of the study: FD, BD, AM
Conception, supervision, and validation of the study: ML
Guarantor of the integrity of the results: CDT
Have approved the final version of the manuscript: all authors

## Acknowledgements

The authors thank Professor Stanislas Pol for his comments on the manuscript and Thomas Leclerc for sharing his expertise on ketamine toxicity.

## Data availability statement

Data available on request.

## Supplementary data

Supplementary data to this article can be found online at https://doi.org/10.1016/j.jhepr.2023.100950.

## References

[1] Matchett G, Gasanova I, Riccio CA, et al. Etomidate versus ketamine for emergency endotracheal intubation: a randomized clinical trial. Intensive Care Med 2022;48:78–91.
[2] Cohen SP, Bhatia A, Buvanendran A, et al. Consensus guidelines on the use of intravenous ketamine infusions for chronic pain from the American society of regional Anesthesia and pain medicine, the American academy of pain medicine, and the American society of anesthesiologists. Reg Anesth Pain Med 2018;43:521–546.
[3] Garber PM, Droege CA, Carter KE. Continuous infusion ketamine for adjunctive analgosedation in mechanically ventilated, critically ill patients. Pharmacotherapy 2019;39:288–296.
[4] Brennan PG, Landry JK, Miles MVP. Intravenous ketamine as an adjunct to procedural sedation during burn wound care and dressing changes. J Burn Care Res 2019;40:246–250.
[5] Chanques G, Constantin JM, Devlin JW, et al. Analgesia and sedation in patients with ARDS. Intensive Care Med 2020;46:2342–2356.
[6] Chu PS, Kwok SC, Lam KM, et al. 'Street ketamine'-associated bladder dysfunction: a report of ten cases. Hong Kong Med J 2007;13:311–313.
[7] Seto WK, Mak SK, Chiu K, et al. Magnetic resonance cholangiogram patterns and clinical profiles of ketamine-related cholangiopathy in drug users. J Hepatol 2018;69:121–128.
[8] Agence nationale de sécurité du médicament et des produits de santé (ANSM). Ketamine: risk of serious liver damage during prolonged use and/or at high doses – information letter to healthcare professionals [Kétamine: risque d'atteintes hépatiques graves lors d'utilisations prolongées et/ou à doses élevées – lettre aux professionnels de santé]. 2017; https://ansm.sante.fr/S-informer/Points-d-information-Points-d-information/Ketamine-risque-d-atteintes-hepatiques-graves-lors-d-utilisations-prolongees-et-ou-a-doses-elevees-Point-d-Information. [Accessed 20 June 2017].
[9] Agence nationale de sécurité du médicament et des produits de santé (ANSM). Ketamine: risk of serious uro-nephrological, endocrinological, and hepatic damages during prolonged use and/or at high doses [Kétamine: risque d'atteintes uro-néphrologiques, endocriniennes et hépatiques graves lors d'utilisations prolongées et/ou à doses élevées]. 2018; https://ansm.sante.fr/informations-de-securite/ketamine-risque-datteintes-uro-nephrologiques-endocriniennes-et-hepatiques-graves-lors-dutilisations-prolongees-et-ou-a-doses-elevees. [Accessed 7 November 2017].
[10] de Tymowski C, Dépret F, Soussi S, et al. Contributing factors and outcomes of burn-associated cholestasis. J Hepatol 2019;71:563–572.
[11] Legrand M, de Tymowski C, Hodjat K, et al. Reply to: "Potential role of ketamine in burn-associated cholestasis". J Hepatol 2019;71:1276–1277.
[12] Soussi S, Dépret F, Benyamina M, et al. Early hemodynamic management of critically ill burn patients. Anesthesiology 2018;129:583–589.
[13] Fawaz R, Baumann U, Ekong U, et al. Guideline for the evaluation of cholestatic jaundice in infants: joint recommendations of the north American society for pediatric gastroenterology, hepatology, and nutrition and the European society for pediatric gastroenterology, hepatology, and nutrition. J Pediatr Gastroenterol Nutr 2017;64:154–168.
[14] Hoofnagle JH, Björnsson ES. Drug-induced liver injury – types and phenotypes. N Engl J Med 2019;38:264–273.
[15] European Association for the Study of the Liver. EASL Clinical Practice Guidelines: management of cholestatic liver diseases. J Hepatol 2009;51:237–267.
[16] Aithal GP, Watkins PB, Andrade RJ, et al. Case definition and phenotype standardization in drug-induced liver injury. Clin Pharmacol Ther 2011;89:806–815.
[17] European Association for the Study of the Liver. EASL Clinical Practice Guidelines: drug-induced liver injury. J Hepatol 2019;70:1222–1261.
[18] Hayashi PH, Lucena MI, Fontana RJ. RECAM: a new and improved, computerized causality assessment tool for DILI diagnosis. Am J Gastroenterol 2022;117:1387–1389.
[19] Hayashi PH, Lucena MI, Fontana RJ, et al. A revised electronic version of RUCAM for the diagnosis of DILI. Hepatology 2022;76:18–31.
[20] Le Gall JR, Lemeshow S, Saulnier F. A new Simplified Acute Physiology Score (SAPS II) based on a European/North American multicenter study. JAMA 1993;270:2957–2963.
[21] Vincent J-L, Moreno R, Takala J, et al. The SOFA (Sepsis-related organ failure assessment) score to describe organ dysfunction/failure: on behalf of the working group on sepsis-related problems of the European society

of intensive care medicine (see contributors to the project in the appendix). Intensive Care Med 1996;22:707–710.
[22] Levey AS, James MT. Acute kidney injury. Ann Intern Med 2017;167:ITC66–ITC80.
[23] Cartotto R, Li Z, Hanna S, et al. The acute respiratory distress syndrome (ARDS) in mechanically ventilated burn patients: an analysis of risk factors, clinical features, and outcomes using the Berlin ARDS definition. Burns 2016;42:1423–1432.
[24] Singer M. The new sepsis consensus definitions (Sepsis-3): the good, the not-so-bad, and the actually-quite-pretty. Intensive Care Med 2016;42:2027–2029.
[25] Hansen BB, Klopfer SO. Optimal full matching and related designs via network flows. J Comput Graph Stat 2006;15:609–627.
[26] Stuart EA, Cole SR, Bradshaw CP, et al. The use of propensity scores to assess the generalizability of results from randomized trials. J R Stat Soc Ser A Stat Soc 2011;174:369–386.
[27] Dundee JW, Fee JP, Moore J, et al. Changes in serum enzyme levels following ketamine infusions. Anaesthesia 1980;35:12–16.
[28] Noppers IM, Niesters M, Aarts LPHJ, et al. Drug-induced liver injury following a repeated course of ketamine treatment for chronic pain in CRPS type 1 patients: a report of 3 cases. Pain 2011;152:2173–2178.
[29] Meunier L, Meszaros M, Pageaux GP. Potential role of ketamine in burn-associated cholestasis. J Hepatol 2019;71:1275.
[30] Knooihuizen SA, Aday A, Lee WM. Ketamine-induced sclerosing cholangitis (KISC) in a critically ill patient with COVID-19. Hepatology 2021;74:519–521.
[31] Keta-Cov research group. Intravenous ketamine and progressive cholangiopathy in COVID-19 patients. J Hepatol 2021;74:1243–1244.
[32] Mallet V, Bock K, Dellagi M, et al. Reply to: "Progressive cholangiopathy in COVID-19 patients: other possible diagnoses than ketamine-induced cholangiopathy should be considered". J Hepatol 2021;75:990–992.
[33] Wendel-Garcia PD, Erlebach R, Hofmaenner DA, et al. Long-term ketamine infusion-induced cholestatic liver injury in COVID-19-associated acute respiratory distress syndrome. Crit Care 2022;26:148.
[34] Hartl L, Haslinger K, Angerer M, et al. Progressive cholestasis and associated sclerosing cholangitis are frequent complications of COVID-19 in patients with chronic liver disease. Hepatology 2022;76:1563–1575.
[35] Horvatits T, Drolz A, Trauner M. Liver injury and failure in critical illness. Hepatology 2019;70:2204–2215.
[36] Kramer L, Jordan B, Druml W, et al. Austrian Epidemiologic Study on Intensive Care ASG. Incidence and prognosis of early hepatic dysfunction in critically ill patients – a prospective multicenter study. Crit Care Med 2007;35:1099–1104.
[37] Kalkan Y, Tomak Y, Altuner D, et al. Hepatic effects of ketamine administration for 2 weeks in rats. Hum Exp Toxicol 2014;33:32–40.
[38] Yu WL, Cho CC, Lung PF, et al. Ketamine-related cholangiopathy: a retrospective study on clinical and imaging findings. Abdom Imaging 2014;39:1241–1246.
[39] Wai MS, Chan WM, Zhang AQ, et al. Long-term ketamine and ketamine plus alcohol treatments produced damages in liver and kidney. Hum Exp Toxicol 2012;31:877–886.
[40] Reich DL, Silvay G. Ketamine: an update on the first twenty-five years of clinical experience. Can J Anaesth 1989;36:186–197.
[41] Licata M, Pierini G, Popoli G. A fatal ketamine poisoning. J Forensic Sci 1994;39:1314–1320.
[42] Ou SH, Wu LY, Chen HY, et al. Risk of renal function decline in patients with ketamine-associated uropathy. Int J Environ Res Public Health 2020;17:7260.
[43] Jensen JS, Peters L, Itenov TS, et al. Biomarker-assisted identification of sepsis-related acute liver impairment: a frequent and deadly condition in critically ill patients. Clin Chem Lab Med 2019;57:1422–1431.
[44] Andonovic M, Traynor JP, Shaw M, et al. Short- and long-term outcomes of intensive care patients with acute kidney disease. EClinicalMedicine 2022;44:101291.
[45] Deniau B, Mallet V, de Tymowski C, et al. Ketamine-based analgosedation for dressing changes in burn patients. Eur J Anaesthesiol 2022;39:845–846.

Journal of Hepatology, Volume *6*

## Supplemental information

# Ketamine restriction correlates with reduced cholestatic liver injury and improved outcomes in critically ill patients with burn injury

**Christian De Tymowski, François Dépret, Emmanuel Dudoignon, Nabila Moreno, Anne-Marie Zagdanski, Kyann Hodjat, Benjamin Deniau, Alexandre Mebazaa, Matthieu Legrand, Vincent Mallet, and for the Keta-Cov research group**

# Ketamine restriction correlates with reduced cholestatic liver injury and improved outcomes in critically ill burn patients

Christian De Tymowski, François Dépret, Emmanuel Dudoignon, Nabila Moreno, Anne-Marie Zagdanski, Kyann Hodjat, Benjamin Deniau, Alexandre Mebazaa, Matthieu Legrand, Vincent Mallet for the Keta-Cov research group

Table of contents

List of investigators ........................................................................................ 2

Fig. S1 ............................................................................................................ 3

Fig. S2 ............................................................................................................ 4

Fig. S3 ............................................................................................................ 5

Table S1 .......................................................................................................... 6

Table S2 .......................................................................................................... 9

Table S3 .......................................................................................................... 11

**List of investigators**

*The Keta-Burn research group*

Christian De Tymowski[1,2,3], François Dépret[1,3,4,5,6], Emmanuel Dudoignon[3], Nabila Moreno[7], Anne-Marie Zagdanski[8], Kyann Hodjat[3], Benjamin Deniau[1,3,4,6], Maxime Coutrot,[3] Mourad Benyamina,[3] Thibault Michel,[3] Alexandru Cupaciu,[3] Alexandre Pharaboz,[3] Maurice Mimoun,[3] Marc Chaouat,[3] Kevin Serror,[3] Benoit Plaud,[3] Maïté Chaussard,[3] Lucie Guillemet,[3] Alexandre Mebazaa[1,3,5,7], Matthieu Legrand[6,9], Vincent Mallet[1,3,10]

1. Université Paris Cité, F-75006, Paris, France
2. Department of Anaesthesiology and Surgical Intensive Care Unit, Groupe Hospitalier Bichat Claude Bernard, DMU PARABOL, Assistance Publique-Hôpitaux de Paris, Paris, France.
3. AP-HP.Nord, Groupe Hospitalier Saint Louis Lariboisière, DMU PARABOL, Département d'anesthésie réanimation et centre de traitement des brûlés, Paris, France
4. Institut National de la Santé et de la Recherche Médicale (INSERM), INSERM UMR-S 942 Mascot, Lariboisière Hospital, Paris, France
5. INI-CRCT Network, Nancy, France
6. FHU PROMICE, Paris, France
7. AP-HP.Nord, Groupe Hospitalier Saint Louis Lariboisière, Laboratoire de Biochimie, Paris, France;
8. AP-HP.Nord, Groupe Hospitalier Saint Louis Lariboisière, Département de radiologie, Paris, France;
9. Department of Anesthesia and Peri-operative Care, Division of Critical Care Medicine, University of California, San Francisco, USA
10. Assistance Publique — Hôpitaux de Paris (AP—HP), Groupe Hospitalier Cochin Port Royal, DMU Cancérologie et spécialités médico-chirurgicales, Service d'Hépatologie, Paris, France

**Supplementary figures**

**Fig. S1: Covariate balance in the propensity matched samples**

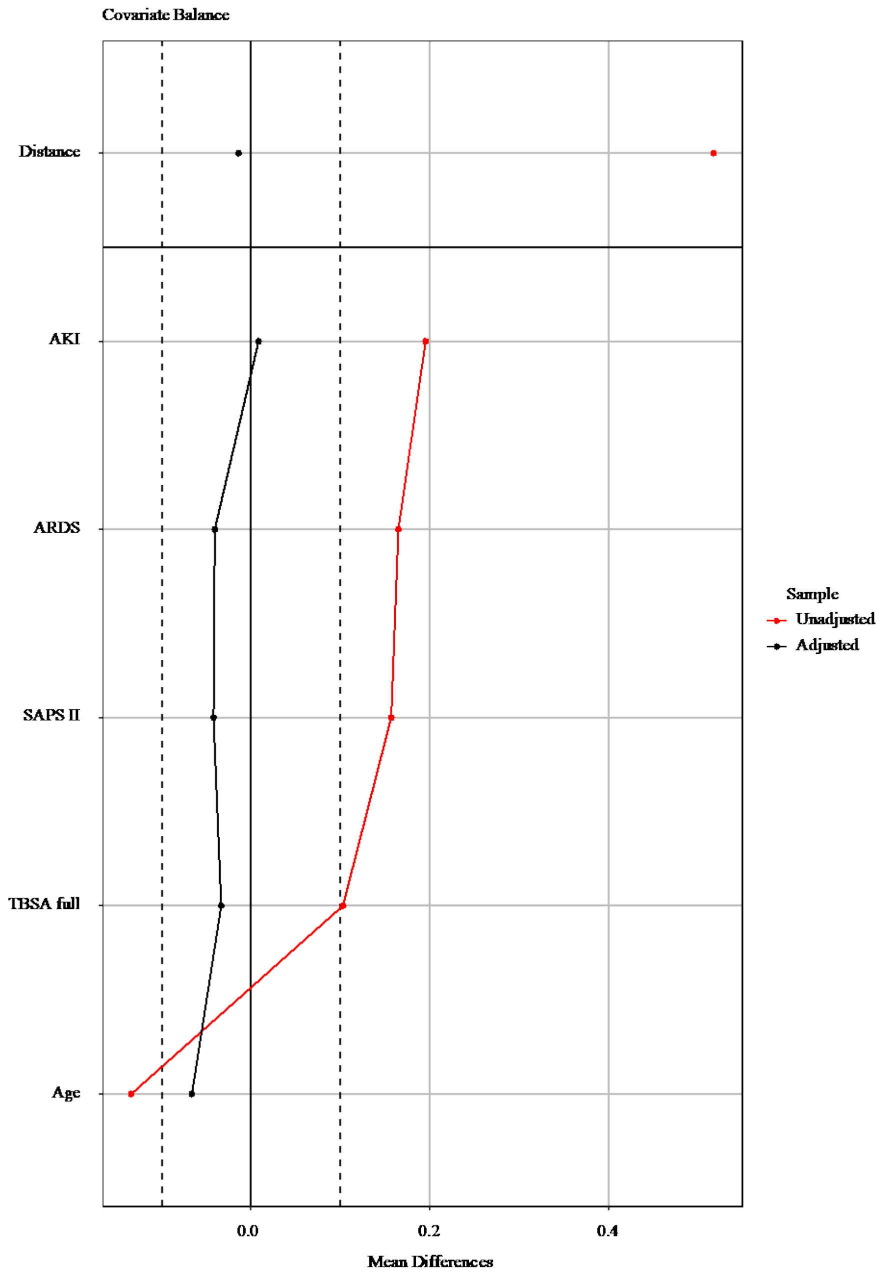


Matching was performed using the MatchIt package (Ho, Imai, King, & Stuart, *2011*) , and covariate balance was assessed using Cobalt Greifer, N. (2023). cobalt: Covariate Balance Tables and Plots. R package version 4.4.1.9003. AKI: acute kidney injury; ARDS: Acute Respiratory Distress Syndrome FTB BSA: full thickness burned surface area; SAPS II: simplified acute physiology score II; SOFA: Sequential Organ Failure Assessment score

## Fig. S2: Liver test evolution over the study period

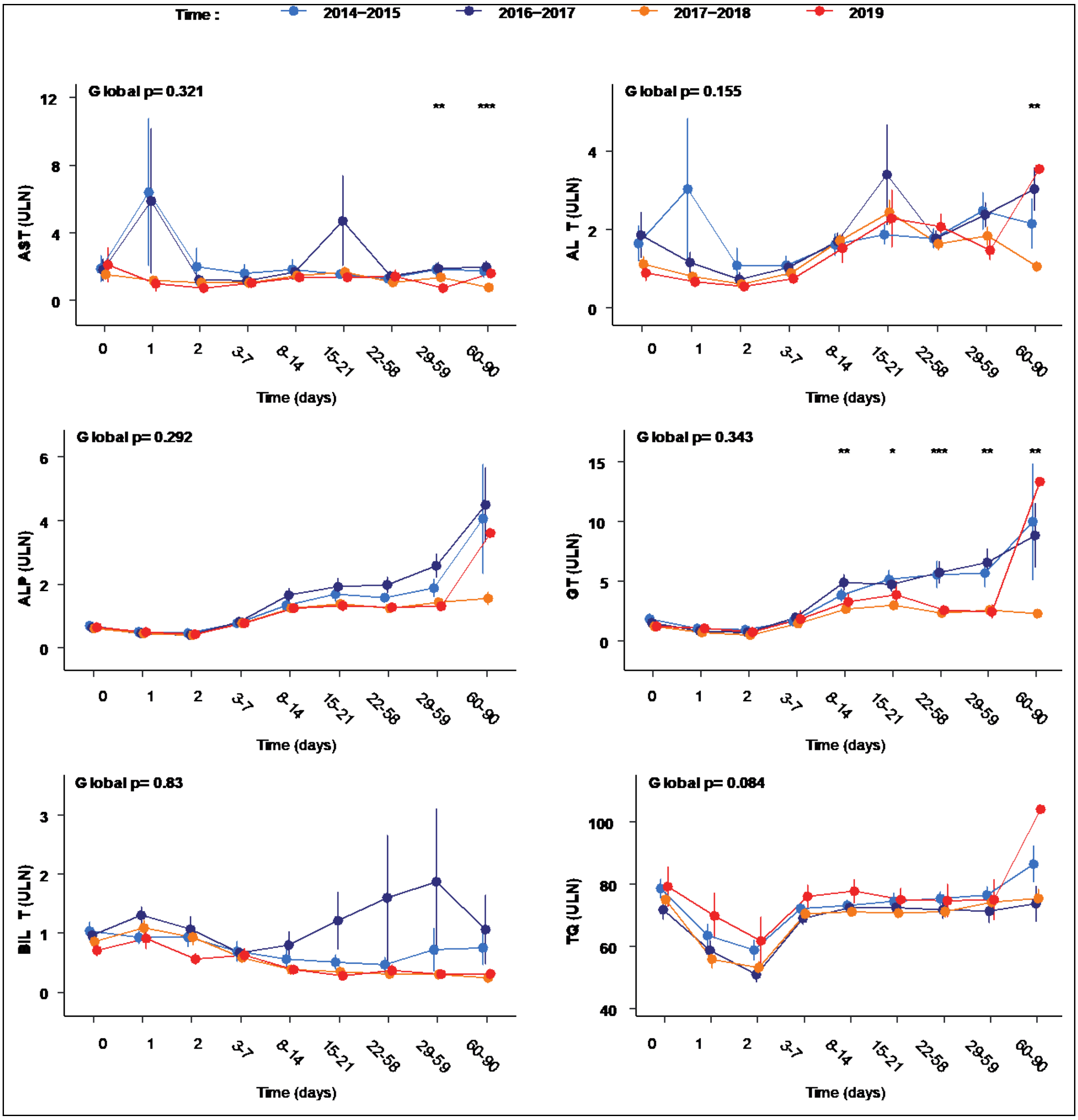


Global p-values were computed with mixed models. The comparison of the four groups at each time point was performed with Kruskal-Wallis tests, with statistical significance indicated as follows: (ns, $p > 0.05$; * $p < 0.05$; ** $p < 0.01$; *** $p < 0.001$; **** $p < 0.0001$). The time periods 2014-2015 and 2016-2017 corresponded to the ketamine-liberal period from December 2014 to the end of March 2017 when ketamine prescription was 'liberally' used for maintenance sedation (≥ 1mg/kg/h). The time periods 2017-2018 and 2019 corresponded to the ketamine-restricted period, from April 2017 to June 2019, when ketamine was only used as a second-line co-analgesic drug with a capped dose (< 0.015mg/kg/h). Note: ALP stands for alkaline phosphatase; ALT for alanine aminotransferase; AST for aspartate aminotransferase; BILT for total bilirubin; and GGT for gamma-glutamyl transferase.

**Fig. S3: Cholangitis in a patient exposed to ≥ 10,000 mg ketamine**

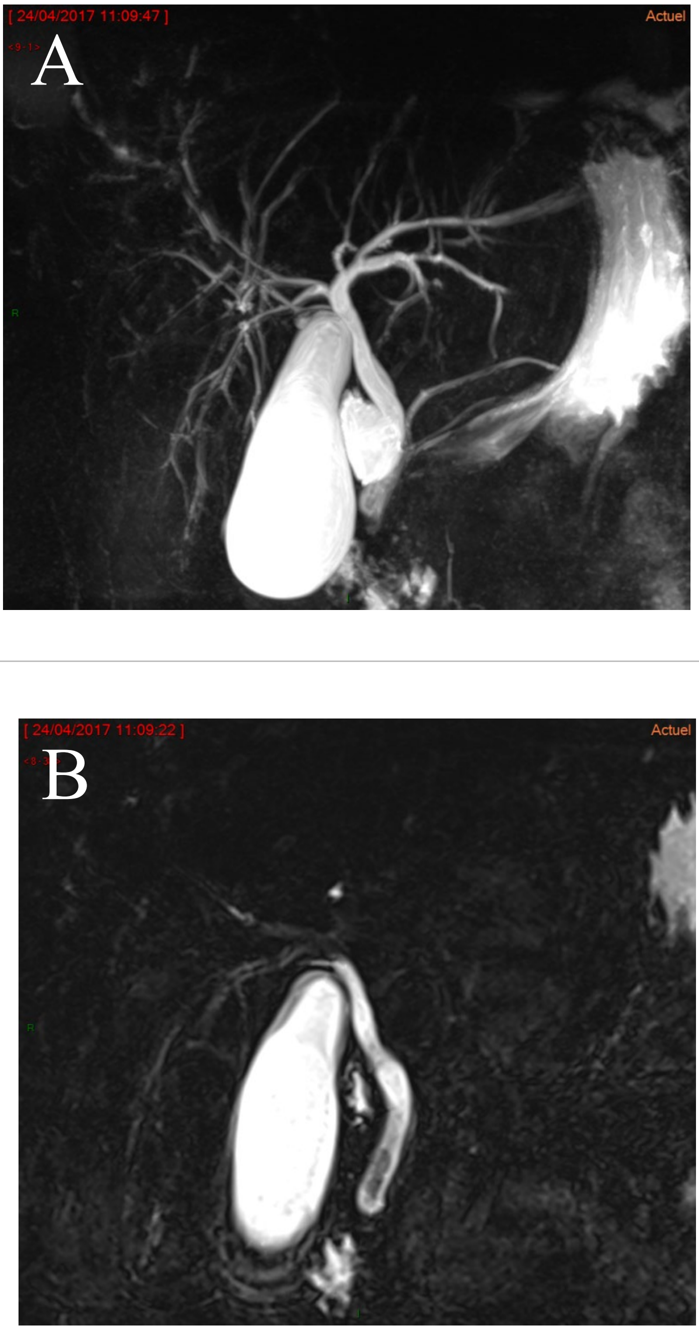


A MR Cholangiopancreatography with a maximum intensity projection (MIP) reconstruction from a 3D thin section showing dilation of the bile ducts;

B Thin sections of the MRI showing biliary casts in the common bile duct

**Table S1: Characteristics of patients with cholestatic liver injury**

| Time Period | Sex | Age (years) | TBSA (%) | ket doses (mg) | Ket days | Time to CLI (days) | Time ket-CLI (days) | Infection | Delay Infection-CLI (days) | Antibiotics | Antifungal | Antiviral | CT-scan | RECAM score | RECAM probability | ALP ≥ grade 3 | Long term liver injury |
|---|---|---|---|---|---|---|---|---|---|---|---|---|---|---|---|---|---|
| **Liberal** | M | 36.2 | 30 | 0 | 0 | 17 | - | y | 5 | Piperacillin Tazobactam | | | Normal | - | - | n | |
| **Liberal** | M | 26.3 | 57 | 1,441 | 5 | 14 | -30 | y | 6 | Piperacillin Tazobactam | | | | -11 | Unlikely | y | |
| **Liberal** | M | 55.3 | 16 | 0 | 0 | 9 | - | n | - | Cefepime | | | | - | - | y | |
| **Liberal** | F | 84 | 59 | 0 | 0 | 1 | - | n | - | | | | | - | - | n | |
| **Liberal** | F | 64.1 | 57 | 17,826 | 46 | 10 | 10 | n | - | Piperacillin Tazobactam | | | Normal | -1 | Possible | y | UPC |
| **Liberal** | M | 43.3 | 31 | 42,206 | 16 | 19 | 18 | y | 9 | | Amphotericin B | | Normal | 3 | Possible | n | UPC |
| **Liberal** | M | 69.9 | 16 | 5,996 | 4 | 19 | 11 | y | 12 | Amoxicillin Clavulanate | | | Normal | 9 | Highly probable | n | |
| **Liberal** | F | 50.7 | 40 | 43,775 | 34 | 47 | 46 | y | 3 | Piperacillin Tazobactam | Amphotericin B | | BDD | 5 | Probable | y | UPC/Biliary sepsis |
| **Liberal** | F | 69.1 | 70 | 29,469 | 13 | 12 | 12 | y | 5 | | Amphotericin B | | Normal | 9 | Highly probable | y | |
| **Liberal** | M | 49.6 | 60 | 21,232 | 6 | 10 | 10 | y | 1 | | Amphotericin B | | Normal | 3 | Possible | n | |
| **Liberal** | M | 47.5 | 62 | 10,722 | 23 | 5 | 5 | y | 1 | Cefepime | | | Normal | 4 | Probable | y | UPC |
| **Liberal** | M | 53.1 | 45 | 16,879 | 11 | 7 | 6 | n | - | | | | Normal | 6 | Probable | n | |
| **Liberal** | M | 40.1 | 55 | 9,451 | 16 | 32 | 31 | y | 1 | Cefepime | | | Normal | 5 | Probable | n | |
| **Liberal** | M | 25.3 | 70 | 28,289 | 32 | 14 | 12 | y | 3 | | Caspofungine | | BDD; Multiple liver abscess | 5 | Probable | y | |
| **Liberal** | M | 57 | 58 | 12,173 | 38 | 68 | 67 | y | 7 | Ceftazidime | | | Normal | 3 | Possible | n | |

| | | | | | | | | | | | | | | | | | |
|---|---|---|---|---|---|---|---|---|---|---|---|---|---|---|---|---|---|
| **Liberal** | M | 56 | 80 | 15,490 | 13 | 8 | 7 | y | 4 | Piperacillin Tazobactam | | | Normal | -4 | Unlikely | y | UPC |
| **Liberal** | M | 51 | 70 | 9,403 | 6 | 9 | 9 | y | 0 | Amoxicillin Clavulanate | | | Liver dysmorp hia | 2 | Possible | y | |
| **Liberal** | F | 25.8 | 65 | 28,279 | 10 | 23 | 23 | y | 7 | Meronem | | | Normal | 3 | Possible | n | UPC |
| **Liberal** | M | 32.7 | 86 | 10,421 | 52 | 11 | 11 | y | 4 | Meronem | | | Normal | 5 | Probable | y | UPC/ Biliary sepsis |
| **Liberal** | M | 68.3 | 20 | 20,121 | 7 | 8 | 3 | y | 0 | Amoxicillin Clavulanate | | | Normal | 2 | Possible | y | |
| **Liberal** | M | 35.9 | 83,5 | 39,135 | 22 | 16 | 16 | y | 4 | | Amphoteric in B | Acyclov ir | Normal | 5 | Probable | n | UPC |
| **Liberal** | F | 68.1 | 37 | 41,339 | 17 | 11 | 10 | y | 6 | Piperacillin Tazobactam | | Acyclov ir | | 7 | Probable | y | |
| **Liberal** | F | 72.3 | 20 | 20 | 1 | 10 | 7 | y | 5 | | | | Normal | 4 | Probable | n | |
| **Liberal** | F | 77.5 | 30 | 17,219 | 29 | 19 | 18 | y | 4 | Piperacillin Tazobactam | | | BDD | 5 | Probable | y | UPC/ Biliary sepsis |
| **Liberal** | F | 55.8 | 35 | 4,525 | 35 | 5 | 4 | n | - | | | | | 0 | Possible | y | UPC |
| **Liberal** | F | 65.2 | 9 | 0 | 0 | 1 | - | n | - | | | | | - | - | n | |
| **Liberal** | F | 26.9 | 50 | 1,132 | 11 | 16 | 16 | y | 6 | Piperacillin Tazobactam | | | Normal | 7 | Probable | y | |
| **Restricte d** | M | 60.6 | 7 | 130 | 3 | 14 | 6 | y | 6 | Amoxicillin Clavulanate | | | BDD | 1 | Possible | n | |
| **Restricte d** | M | 55.7 | 39 | 0 | 0 | 37 | - | y | 8 | Piperacillin Tazobactam | Fluconazole | | Normal | - | - | n | |
| **Restricte d** | M | 26.8 | 72 | 130 | 4 | 4 | -3 | n | 2 | Piperacillin Tazobactam | | | Normal | -11 | Unlikely | y | |
| **Restricte d** | M | 47.7 | 30 | 23 | 2 | 13 | -4 | y | 3 | Cefepime | | | | -11 | Unlikely | n | |
| **Restricte d** | M | 29.3 | 20 | 250 | 4 | 8 | 6 | y | 3 | trimethoprim sulfamethoxa zole | | | Normal | 8 | Highly probable | n | |
| **Restricte d** | F | 49.4 | 7 | 50 | 1 | 4 | 0 | y | 5 | Amoxicillin Clavulanate | | | Normal | -1 | Possible | n | |

| | | | | | | | | | | | | | | | | | |
|---|---|---|---|---|---|---|---|---|---|---|---|---|---|---|---|---|---|
| **Restricted** | M | 16.6 | 38 | 0 | 0 | 29 | - | y | 10 | Cefotaxime | | | | - | - | n | |

Note: BDD: bile duct dilatation; CLI: cholestatic liver injury; Ket: Ketamine; n=no, UPC: unexplained prolonged cholestatic liver injury; y=yes

| Table S2: Characteristics of patients by day-90 mortality | | | | |
|---|---|---|---|---|
| **Characteristic** | Overall, N = 278 (100%)[1] | Day-90 mortality | | p-value[2] |
| | | No death, N = 221 (79%)[1] | Death, N = 57 (21%)[1] | |
| **Ketamine dose reduction period** | | | | 0.031 |
| Before reduction | 155 (55.8%) | 116 (52.5%) | 39 (68.4%) | |
| After reduction | 123 (44.2%) | 105 (47.5%) | 18 (31.6%) | |
| **Cholestatic liver injury** | 34 (12.2%) | 20 (9.0%) | 14 (24.6%) | 0.001 |
| **Grade ≥ 3 ALP elevation** | 25 (9.0%) | 17 (7.7%) | 8 (14.0%) | 0.14 |
| **DILI cholestasis** | 106 (38.1%) | 85 (38.5%) | 21 (36.8%) | 0.8 |
| **Cholestasis** | 174 (62.6%) | 133 (60.2%) | 41 (71.9%) | 0.10 |
| **Male sex** | 176 (63.3%) | 144 (65.2%) | 32 (56.1%) | 0.2 |
| **Age, years** | 50.7 (31.5, 67.3) | 45.7 (29.7, 61.4) | 65.2 (51.0, 81.5) | <0.001 |
| **Body mass index, Kg/m2** | 25.1 (22.9, 28.7) | 24.8 (22.9, 28.1) | 26.1 (22.5, 29.8) | 0.3 |
| **Electrical burn** | 14 (5.0%) | 14 (6.3%) | 0 (0.0%) | 0.081 |
| **Thermal burn** | 267 (96.0%) | 210 (95.0%) | 57 (100.0%) | 0.13 |
| **Body surface area burned, %** | 28.3 (20.0, 45.0) | 25.0 (20.0, 40.0) | 33.5 (20.0, 65.0) | 0.005 |
| **Full-thickness body surface area burned, %** | 15.0 (5.0, 27.0) | 11.5 (4.0, 24.3) | 23.0 (14.0, 54.0) | <0.001 |
| **Inhalation injury** | 91 (32.7%) | 56 (25.3%) | 35 (61.4%) | <0.001 |
| **ABSI** | 8.0 (6.0, 10.0) | 7.0 (6.0, 9.0) | 10.0 (8.0, 12.0) | <0.001 |
| **SAPS II** | 29.0 (19.0, 41.0) | 25.0 (16.8, 35.3) | 47.0 (35.0, 63.0) | <0.001 |
| **SOFA** | 2.0 (0.0, 6.0) | 1.0 (0.0, 4.0) | 7.0 (3.0, 10.3) | <0.001 |
| **Volume expansion with crystalloid fluids, ml/kg/%** | 4.0 (2.4, 5.1) | 3.9 (2.2, 5.0) | 4.9 (3.2, 6.6) | 0.017 |
| **Vasopressors administration at admission** | 107 (38.8%) | 63 (28.8%) | 44 (77.2%) | <0.001 |
| **Length of vasopressors infusion, d** | 1.0 (0.0, 3.0) | 0.0 (0.0, 2.0) | 2.0 (1.0, 12.0) | <0.001 |
| **Mechanical ventilation at admission** | 172 (61.9%) | 125 (56.6%) | 47 (82.5%) | <0.001 |
| **Duration of mechanical ventilation, d** | 3.0 (0.0, 30.8) | 3.0 (0.0, 33.0) | 4.0 (1.0, 21.0) | 0.3 |
| **Initial AST level, x ULN** | 0.9 (0.7, 1.4) | 0.9 (0.7, 1.4) | 0.9 (0.8, 1.6) | 0.7 |
| **Initial ALT level, x ULN** | 0.7 (0.5, 1.1) | 0.7 (0.5, 1.0) | 0.7 (0.5, 1.3) | 0.3 |
| **Initial GGT level, x ULN** | 0.6 (0.4, 1.4) | 0.6 (0.4, 1.3) | 0.6 (0.4, 1.9) | 0.2 |
| **Initial ALP level, x ULN** | 0.6 (0.5, 0.7) | 0.6 (0.5, 0.7) | 0.6 (0.5, 0.9) | 0.003 |
| **Initial BILT level, x ULN** | 0.7 (0.4, 1.1) | 0.6 (0.4, 1.0) | 1.0 (0.6, 1.3) | <0.001 |
| **Initial prothrombin ratio, %** | 79.0 (64.0, 89.0) | 81.0 (71.0, 90.0) | 55.5 (41.0, 83.5) | <0.001 |
| **Initial serum creatinine level, µmol/L** | 72.0 (57.7, 94.0) | 68.8 (56.9, 85.5) | 101.0 (69.0, 120.0) | <0.001 |
| **Enteral nutrition** | 187 (67.3%) | 150 (67.9%) | 37 (64.9%) | 0.7 |
| **Parenteral nutrition** | 18 (6.5%) | 10 (4.5%) | 8 (14.0%) | 0.015 |
| **Acute kidney injury** | 95 (34.2%) | 47 (21.3%) | 48 (84.2%) | <0.001 |
| **Renal replacement therapy** | 39 (14.0%) | 14 (6.3%) | 25 (43.9%) | <0.001 |
| **Acute respiratory distress syndrome** | 65 (23.4%) | 32 (14.5%) | 33 (57.9%) | <0.001 |
| **Septic shock** | 76 (27.3%) | 48 (21.7%) | 28 (49.1%) | <0.001 |
| **Total ketamine exposure, mg** | 41.8 (0.0, 635.8) | 50.0 (0.0, 556.9) | 10.0 (0.0, 2,540.0) | 0.6 |
| **Ketamine dose distribution, mg** | | | | 0.031 |
| [-Inf,0] | 106 (38.1%) | 79 (35.7%) | 27 (47.4%) | |
| (0,1,000] | 105 (37.8%) | 93 (42.1%) | 12 (21.1%) | |
| (1,000,10,000] | 33 (11.9%) | 25 (11.3%) | 8 (14.0%) | |
| (10,000, Inf] | 34 (12.2%) | 24 (10.9%) | 10 (17.5%) | |
| **Length of ketamine infusion, d** | 1.0 (0.0, 6.0) | 1.0 (0.0, 6.0) | 1.0 (0.0, 4.0) | 0.13 |

| | | | | |
|---|---|---|---|---|
| **Time to ketamine exposure> 10,000 mg** | 6.0 (5.0, 8.8) | 6.0 (6.0, 11.0) | 5.0 (3.5, 6.0) | 0.045 |
| **Number of patients without ketamine infusion** | 107 (38.5%) | 80 (36.2%) | 27 (47.4%) | 0.12 |
| **Total midazolam exposure, mg** | 5.4 (0.0, 457.3) | 5.0 (0.0, 419.2) | 55.4 (0.0, 532.2) | 0.7 |
| **Length of midazolam infusion, d** | 1.0 (0.0, 4.0) | 1.0 (0.0, 4.0) | 1.0 (0.0, 4.0) | 0.9 |
| **Number of patients without midazolam infusion** | 121 (43.5%) | 96 (43.4%) | 25 (43.9%) | >0.9 |
| **Total sufentanil exposure, µg** | 169.9 (0.0, 2,527.8) | 159.3 (0.0, 2,610.3) | 269.3 (0.0, 1,421.5) | 0.8 |
| **Length of sufentanil infusion, d** | 3.0 (0.0, 14.0) | 3.0 (0.0, 16.0) | 2.0 (0.0, 8.0) | 0.3 |
| **Number of patients without sufentanil infusion** | 77 (27.7%) | 61 (27.6%) | 16 (28.1%) | >0.9 |
| **Length of stay in the ICU** | 29.5 (14.3, 48.0) | 32.0 (19.0, 53.0) | 10.0 (1.0, 31.0) | <0.001 |

Cholestasis was serum ALP ≥ 1.5 x ULN with GGT ≥ 3 x ULN; cholestatic liver injury was serum ALP ≥ 1.5 x ULN with GGT ≥ 3 x ULN and BILT > 1 x ULN; DILI cholestasis was serum ALP level ≥ 2 x ULN and serum GGT level ≥ 1 x ULN; Grade 3 or higher ALP elevation was serum ALP ≥ 5 x ULN. The SAPS II ranges from 0 to 163, with higher scores indicating greater severity of illness. The ABSI ranges from 0 to 18, with higher scores indicating a greater probability of death after burn injury. The SOFA ranges from 0 to 24 with higher scores indicate more severe organ failure. ABSI: abbreviated burn severity index; ALP: alkaline phosphatase; ALT: alanine aminotransferase ; AST: aspartate aminotransferase; BILT: total bilirubin; DILI: Drug induced liver injury; GGT: gamma-glutamyl transferase; Inf= Infinite; SAPS II: simplified acute physiology score II; SOFA: Sequential Organ Failure Assessment score; ULN : upper limit of normal

1 n (%); Median (IQR)

2 Pearson's Chi-squared test; Wilcoxon rank sum test; Fisher's exact test

| Table S3: Characteristics of ARDS patients by time period | | | | |
|---|---|---|---|---|
| **Characteristic** | **Overall**, N = 65[1] | **Before ketamine reduction**, n = 34[1] | **After ketamine reduction**, n = 31[1] | p-value[2] |
| **Cholestatic liver injury** | 21 (32.3%) | 16 (47.1%) | 5 (16.1%) | 0.008 |
| **Grade = 3 ALP elevation** | 15 (23.1%) | 12 (35.3%) | 3 (9.7%) | 0.014 |
| **DILI cholestasis** | 37 (56.9%) | 21 (61.8%) | 16 (51.6%) | 0.409 |
| **Cholestasis** | 49 (75.4%) | 27 (79.4%) | 22 (71.0%) | 0.430 |
| **Male sex** | 37 (56.9%) | 19 (55.9%) | 18 (58.1%) | 0.859 |
| **Age, years** | 55.3 (39.3-68.1) | 57.2 (44.7-68.9) | 51.3 (34.9-63.3) | 0.163 |
| **Body mass index, Kg/m2** | 26.1 (22.5-29.4) | 26.7 (22.9-29.3) | 26.1 (22.5-29.0) | 0.622 |
| **Electrical burn** | 1 (1.5%) | 0 (0.0%) | 1 (3.2%) | 0.477 |
| **Thermal burn** | 64 (98.5%) | 34 (100.0%) | 30 (96.8%) | 0.477 |
| **Body surface area burned, %** | 40.0 (25.0-60.0) | 39.0 (21.2-59.5) | 45.0 (30.0-63.0) | 0.747 |
| **Full-thickness body surface area burned, %** | 23.0 (12.0-50.0) | 24.5 (13.2-40.8) | 21.0 (10.8-52.0) | 0.833 |
| **Inhalation injury** | 45 (69.2%) | 25 (73.5%) | 20 (64.5%) | 0.432 |
| **ABSI** | 10.0 (8.0-12.0) | 10.0 (8.0-11.8) | 10.0 (7.5-12.0) | 0.963 |
| **SOFA** | 8.0 (4.0-9.0) | 7.5 (5.0-9.0) | 8.0 (3.8-9.2) | 0.775 |
| **Volume expansion with crystalloid fluids, ml/kg/%** | 4.8 (3.9-6.7) | 5.1 (3.9-6.4) | 4.4 (3.9-7.3) | 0.727 |
| **Vasopressors administration at admission** | 46 (71.9%) | 27 (79.4%) | 19 (63.3%) | 0.153 |
| **Length of vasopressors infusion, d** | 4.0 (1.0-13.0) | 7.5 (1.0-17.5) | 3.0 (1.0-7.5) | 0.101 |
| **Mechanical ventilation at admission** | 63 (96.9%) | 32 (94.1%) | 31 (100.0%) | 0.493 |
| **Duration of mechanical ventilation, d** | 24.0 (7.0-50.0) | 21.5 (4.8-36.8) | 27.0 (10.5-62.0) | 0.438 |
| **Initial AST level, x ULN** | 1.1 (0.7-1.7) | 1.1 (0.8-1.2) | 1.5 (0.5-2.1) | 0.689 |
| **Initial ALT level, x ULN** | 0.7 (0.5-1.1) | 0.7 (0.5-1.1) | 0.7 (0.6-1.0) | 0.856 |
| **Initial GGT level, x ULN** | 0.6 (0.3-1.4) | 0.5 (0.4-1.7) | 0.8 (0.3-1.4) | 0.908 |
| **Initial ALP level, x ULN** | 0.6 (0.5-0.8) | 0.6 (0.5-0.9) | 0.6 (0.5-0.8) | 0.220 |
| **Initial BILT level, x ULN** | 0.7 (0.4-1.2) | 0.8 (0.4-1.3) | 0.7 (0.4-1.0) | 0.270 |
| **Initial prothrombin ratio, %** | 67.0 (51.0-82.0) | 64.0 (50.0-82.0) | 69.5 (58.2-81.0) | 0.638 |
| **Initial serum creatinine level, µmol/L** | 88.2 (67.3-118.0) | 91.6 (67.3-118.9) | 85.3 (67.5-115.1) | 0.692 |
| **Enteral nutrition** | 52 (80.0%) | 28 (82.4%) | 24 (77.4%) | 0.619 |
| **Parenteral nutrition** | 9 (13.8%) | 7 (20.6%) | 2 (6.5%) | 0.153 |
| **Acute kidney injury** | 46 (70.8%) | 26 (76.5%) | 20 (64.5%) | 0.290 |
| **Renal replacement therapy** | 27 (41.5%) | 19 (55.9%) | 8 (25.8%) | 0.014 |
| **Acute respiratory distress syndrome** | 65 (100.0%) | 34 (100.0%) | 31 (100.0%) | |
| **Sepsis** | 47 (72.3%) | 26 (76.5%) | 21 (67.7%) | 0.432 |
| **Number of sepsis** | 2.0 (1.0-3.0) | 2.0 (1.0-3.0) | 2.0 (1.0-4.0) | 0.350 |
| **Septic shock** | 37 (56.9%) | 20 (58.8%) | 17 (54.8%) | 0.746 |
| **Number of septic shock** | 2.0 (1.0-3.0) | 1.0 (1.0-2.0) | 2.0 (1.0-3.2) | 0.209 |

| | | | | |
|---|---|---|---|---|
| **Total ketamine exposure, mg** | 75.0 (0.0-10,421.1) | 8,623.8 (18.4-20,954.4) | 0.0 (0.0-82.5) | <0.001 |
| **Length of stay in the ICU** | 32.0 (9.0-73.0) | 28.5 (6.5-49.0) | 52.0 (14.0-91.0) | 0.166 |
| **28-day mortality** | 26 (40.0%) | 17 (50.0%) | 9 (29.0%) | 0.085 |
| **90-day mortality** | 33 (50.8%) | 23 (67.6%) | 10 (32.3%) | 0.004 |
| Cholestasis was serum ALP ≥ 1.5 x ULN with GGT ≥ 3 x ULN; cholestatic liver injury was serum ALP ≥ 1.5 x ULN with GGT ≥ 3 x ULN and BILT > 1 x ULN; DILI cholestasis was serum ALP level ≥ 2 x ULN and serum GGT level ≥ 1 x ULN; Grade 3 or higher ALP elevation was serum ALP ≥ 5 x ULN. The SAPS II ranges from 0 to 163, with higher scores indicating greater severity of illness. The ABSI ranges from 0 to 18, with higher scores indicating a greater probability of death after burn injury. The SOFA ranges from 0 to 24 with higher scores indicate more severe organ failure. ABSI: abbreviated burn severity index; ALP: alkaline phosphatase; ALT: alanine aminotransferase ; AST: aspartate aminotransferase; BILT: total bilirubin; DILI: Drug induced liver injury; GGT: gamma-glutamyl transferase; Inf= Infinite; SAPS II: simplified acute physiology score II; SOFA: Sequential Organ Failure Assessment score; ULN : upper limit of normal | | | | |
| 1 n (%); Median (25%-75%) | | | | |
| 2 Pearson's Chi-squared test; Wilcoxon rank sum exact test; Wilcoxon rank sum test; Fisher's exact test | | | | |
